# Hypovirus‐Induced Phosphorylation of CpIre1 Modulates Unfolded Protein Response and Virulence in *Cryphonectria parasitica*

**DOI:** 10.1111/mpp.70227

**Published:** 2026-02-15

**Authors:** Lijiu Zhao, Feng Wang, Fengyue Chen, Suzhen Su, Jinfeng Qiu, Baoshan Chen, Ru Li

**Affiliations:** ^1^ State Key Laboratory for Conservation and Utilization of Subtropical Agro‐Bioresources, College of Life Science and Technology Guangxi University Nanning China; ^2^ College of Agriculture, Guangxi Key Laboratory of Sugarcane Biology Guangxi University Nanning China

**Keywords:** CpIre1, *Cryphonectria parasitica*, hypovirus, phosphorylation, unfolded protein response, virulence

## Abstract

The chestnut blight fungus *Cryphonectria parasitica* and its hypovirus constitute a valuable model for investigating fungal pathogenesis and cross‐kingdom virus–host interplay. To investigate how hypovirus regulates protein function at the phosphorylation level in *C. parasitica* , we performed a comparative phosphoproteomic analysis in the fungus with or without Cryphonectria hypovirus 1 (CHV1) infection. Comparative profiling between the wild‐type (EP155) and hypovirus‐infected (EP155/CHV1‐EP713) strains revealed 700 differentially phosphorylated sites (174 upregulated, 526 downregulated). Among these, the serine 896 and 897 sites on the endoplasmic reticulum (ER) stress‐sensing protein CpIre1 drew our particular attention, as hypovirus‐induced phosphorylation targets. Western blot analysis showed that virus‐encoded p29, p40, and p48 proteins could promote CpIre1 phosphorylation. Site‐specific mutagenesis revealed that Ser‐896 and Ser‐897 are essential for CpIre1 phosphorylation, which regulates fungal phenotypic traits, virulence, and stress tolerance in *C. parasitica.* Reverse‐transcription‐quantitative PCR analysis of the ER stress marker genes *CpHac1* and *CpBip1* confirmed that CpIre1 and its phosphorylation are essential for a functional ER stress response. Notably, hypovirus replication was significantly impaired in phospho‐deficient *CpIre1* mutants, showing about 40% reduction in viral RNA accumulation, whereas phospho‐mimic mutants maintained wild‐type levels of viral RNA. This indicates that efficient hypovirus accumulation requires functional phosphorylation of CpIre1. Our findings demonstrate that hypovirus‐induced phosphorylation of CpIre1 modulates fungal ER homeostasis, pathogenicity, and viral RNA accumulation, thereby revealing a mechanism through which the virus reprogrammes its host via targeted post‐translational modification.

## Graphical abstract

Hypovirus‐induced phosphorylation of CpIre1 modulates the endoplasmic reticulum stress response in *Cryphonectria parasitica*.

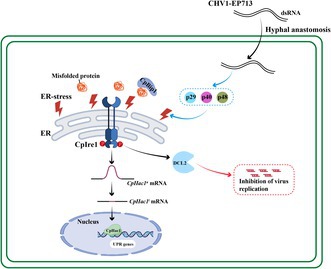

## 1 Introduction

*Cryphonectria parasitica* is the causal agent of chestnut blight (Anagnostakis 1982). *C. parasitica* is capable of supporting the replication of diverse mycoviruses, making it an ideal model for investigating virus–host interactions and fungal pathogenesis (Eusebio‐Cope et al. 2015). Infection by hypoviruses, a group of single‐stranded, positive‐sense RNA viruses, attenuates fungal phenotypes including growth rate, pigmentation, asexual sporulation, male sterility, and virulence (Dawe and Nuss 2001; Nuss 2005). It has been known that these phenotypic changes in the host are associated with the extensive alterations in gene transcription, protein translation and modification, metabolome, and methylation profiles (Allen et al. 2003; Chun et al. 2020; Dawe et al. 2009; Li et al. 2018; Shang et al. 2008). Although several protein kinases in the fungal signal transduction pathway have been shown to be regulated by the hypovirus (Heimel et al. 2013; Miyazaki et al. 2013), comprehensive phosphoproteome analysis of *C. parasitica* has not been reported.

Newly synthesised proteins often undergo post‐translational modifications (PTMs) to become fully functional (Silva‐Sanchez et al. 2015). Although more than 400 types of PTMs have been identified, protein phosphorylation is by far the most common type of PTM in eukaryotes (Ozlu et al. 2010; Ramazi and Zahiri 2021), mediated by kinases and phosphatases to regulate cellular homeostasis and signal transduction (Andreeva and Kutuzov 2008; Manning et al. 2002). Phosphorylation controls a variety of cellular processes, such as metabolism, cell cycle progression, transcription, mating, filamentation, cell wall synthesis, and maintenance of cell integrity (Andrews and Stark 2000; Leach and Brown 2012; Manning et al. 2002; Winkler et al. 2002).

Phosphoproteomics has been employed to investigate phosphorylation changes during various developmental processes, stress responses, and pathogenicity in fungi (Martínez‐Montañés et al. 2020; Ribeiro et al. 2017). For example, in *Saccharomyces cerevisiae* and *Aspergillus fumigatus* , key phosphorylation events regulate stress responses and maintain fungal cell wall integrity (Kanshin et al. 2015; Mattos et al. 2020). In *Neurospora crassa*, phosphorylation of timers was found to be essential in the regulation of fungal biological rhythms (Liu et al. 2000). Additionally, comparative phosphoproteome analyses of proteins responsive to *Magnaporthe oryzae* in susceptible and resistant rice cultivars have revealed key phosphorylation events (e.g., OsMPK6 Thr202/Tyr204 and PBZ1 Ser102) that activate salicylic acid signalling and enhance cell wall fortification, providing mechanistic insights into fungal pathogenicity and host resistance (Li et al. 2015). In *Candida albicans* , dynamic alterations in phosphoprotein abundance during hyphal morphogenesis have been implicated in fungal virulence and morphological transitions (Ghorai et al. 2018). Despite these advances, virus‐mediated phosphorylation regulation in fungi remains underexplored (Jones et al. 2006).

The unfolded protein response (UPR) is a conserved eukaryotic mechanism that alleviates endoplasmic reticulum (ER) stress and facilitates cellular adaptation to various environmental challenges (Ellgaard and Helenius 2003). In animals, the UPR is mediated by three distinct signalling pathways: Inositol‐Requiring Enzyme 1 (IRE1), PKR‐like ER kinase (PERK), and Activating Transcription Factor 6 (ATF6) (Ron and Walter 2007), while fungi primarily rely on the IRE1‐Hac1/Xbp1 pathway. ER stress induces IRE1 oligomerisation and trans‐autophosphorylation, then activating its RNase domain. The activated IRE1 cleaves the *HAC1* mRNA (or *XBP1* in some species), producing an active transcription factor that drives transcription of genes encoding ER chaperones (e.g., BiP1), protein translocation and ER‐associated degradation components. This pathway is essential for stress adaptation, virulence, and secretory capacity in fungi, differing from mammals mainly in its target mRNA (*HAC1* vs. *XBP1*) (Hernández‐Elvira et al. 2018).

In this study, we employed tandem mass tag (TMT) LC–MS/MS analysis to investigate the effect of hypovirus infection on the phosphorylated proteins of *C. parasitica* . The upregulated phosphorylated level of *C. parasitica* IRE1 (CpIre1), a crucial sensor in the UPR, was further confirmed through western blot analysis. Similarly, three important viral proteins p29, p40, and p48 were able to induce the enhanced phosphorylation of CpIre1. Site‐directed mutagenesis assays validated the functional importance of the Ser‐896 and Ser‐897 phosphorylation sites on CpIre1 in *C. parasitica* and hypovirus RNA accumulation. Overall, our findings provide novel insights into the roles and mechanisms of hypovirus‐induced CpIre1 phosphorylation in fungi.

## 2 Results

### 2.1 Characterizations of the Global Phosphoproteome of *C. parasitica*

**FIGURE 1:**
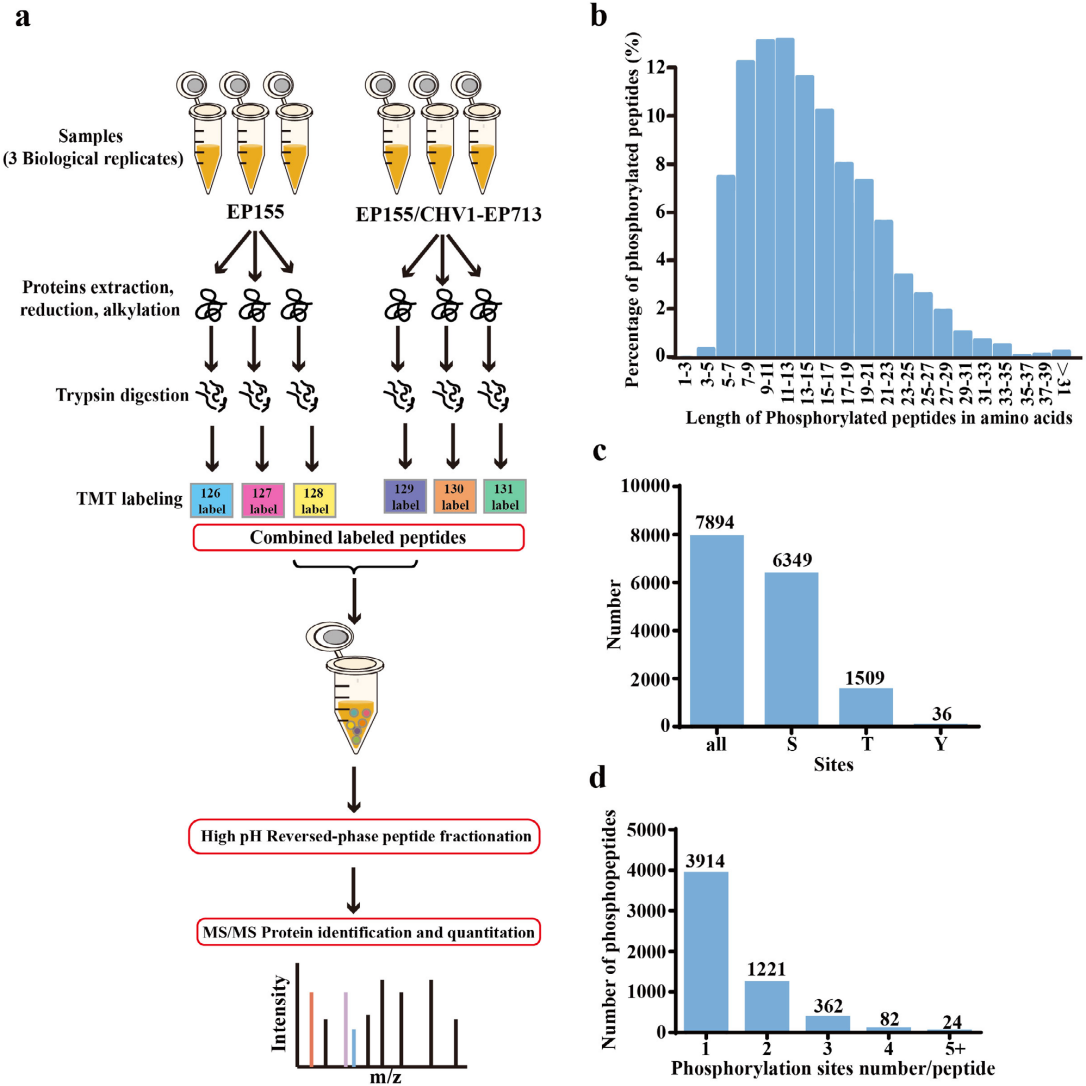
Comprehensive identification of phosphorylation sites and proteins in *Cryphonectria parasitica*. (a) Schematic overview of the workflow used for identifying phosphopeptides and phosphoproteins. (b) Length distribution of detected phosphorylated peptides. (c) Distribution of phosphorylation amino acids of *C. parasitica* . S: serine; T: threonine; Y: tyrosine. (d) Frequency distribution of peptides with single and multiple phosphorylation sites.

To examine the potential impact of hypovirus infection on the global phosphorylation protein level of *C. parasitica* , we employed a TMT‐based quantitative proteomic approach. This methodology combined Fe‐NTA phosphopeptides enrichment with liquid chromatography–tandem mass spectrometry (LC–MS/MS) to analyse the wild‐type strain EP155 and its isogenic Cryphonectria hypovirus 1 (CHV1)‐infected strain EP155/CHV1‐EP713 (Figure 1a). The mass errors of identified peptides were checked to validate the MS data. The distribution of mass error was near 0 and most were less than 6 ppm, indicating that the data met the requirement (Figure S1). Our results revealed the presence of 7894 phosphorylation sites on 5603 peptides, which were distributed among 1819 unique proteins (Table S1).

The majority of peptides ranged from 7 to 19 amino acids in length, consistent with the properties of tryptic peptides (Figure 1b). Among these phosphorylation sites, there were 6349 serine sites, 1509 threonine sites, and 36 tyrosine sites (Figure 1c). Among the 5603 phosphorylated peptides, 3914 contained one phosphorylation site, and most of the other 1689 phosphorylated peptides contained two to four sites (Figure 1d).

By analysing the number of peptides associated with different phosphorylation motifs (Figure S2 and Table S2), Ser‐Pro sequence (“S‐P” motif) emerged as the most frequently phosphorylated, producing 403 phosphorylated peptides, followed by the “RS” (Arg‐Ser motif) and “SS” (Ser‐Ser motif) with 279 and 241 peptides, respectively. In contrast, motifs like “GT_P” (Gly‐Thr‐Pro motif) and “KR_S” (Lys‐Arg‐Ser motif) had significantly less presentation.

### 2.2 Bioinformatic Analysis of Differentially Phosphorylated Proteins

To remove the impact of protein‐level changes on differential phosphorylation analysis, we performed a correlation analysis between the phosphoproteomic data and the corresponding proteomic data available in our laboratory (PRIDE accession: PXD073191). By quantifying phosphosite intensities and normalising them to the abundance of their protein levels, we identified a total of 700 significantly regulated phosphorylation events, including 174 upregulated and 526 downregulated events in EP713 relative to EP155 (Table S3). To functionally understand these phosphorylation‐state changes, we next used the normalised up‐ and downregulated phosphosites to perform GO terms and KEGG pathways.

**FIGURE 2:**
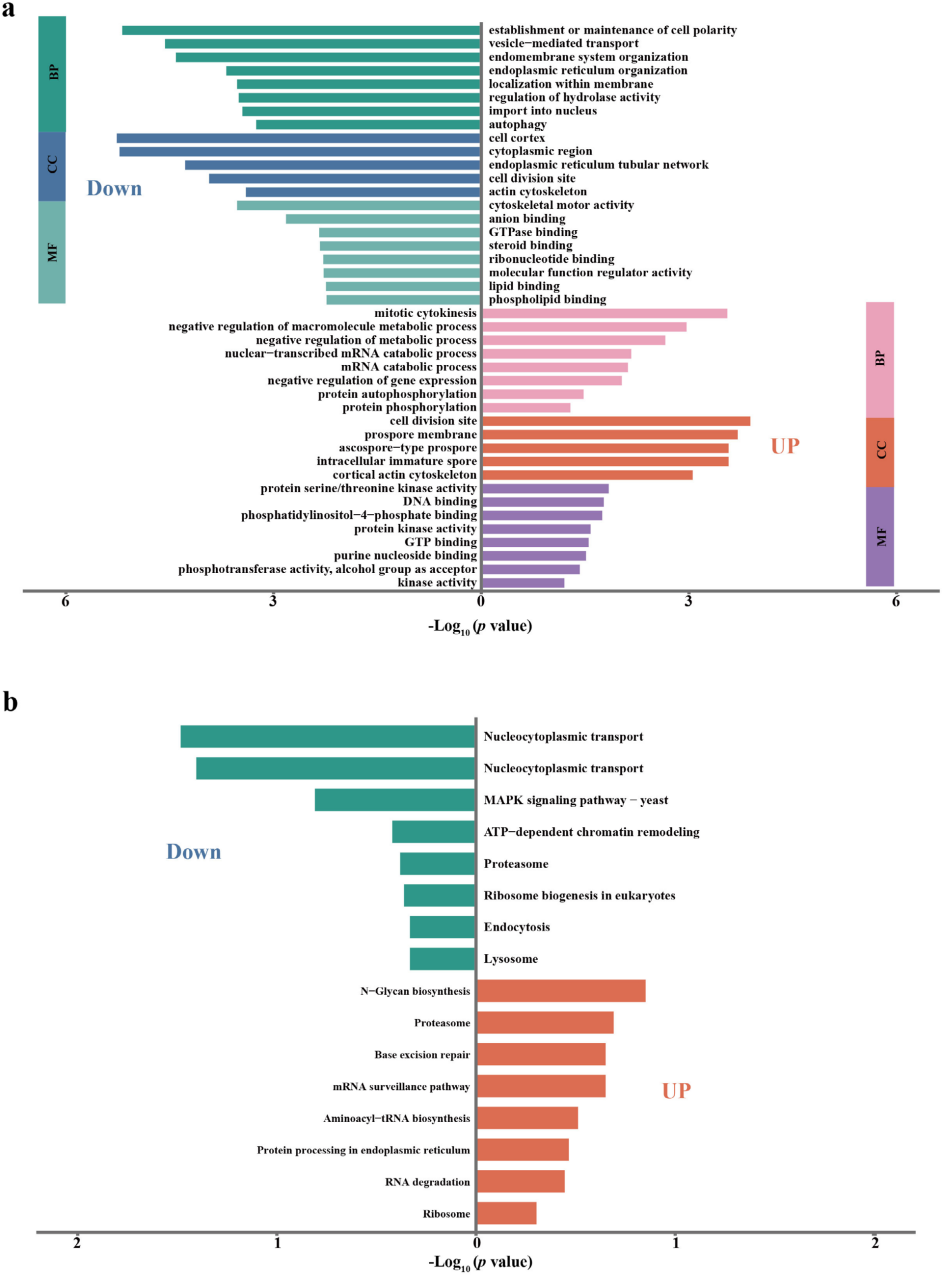
Enrichment analysis of differentially expressed phosphorylated proteins between *Cryphonectria parasitica* wild‐type strain EP155 and Cryphonectria hypovirus 1‐infected strain EP155/CHV1‐EP713. (a) Gene Ontology (GO) enrichment analysis. (b) KEGG pathway enrichment analysis.

As shown in Figure 2a, the normalised downregulated phosphosites were predominantly mapped to processes that coordinate the endomembrane system and intracellular trafficking, including establishment/maintenance of cell polarity, vesicle‐mediated transport, endomembrane and ER organisation, and autophagy. In parallel, the dominant enriched cellular components for the downregulated set included the cell cortex, cytoplasmic region, ER (including the tubular network), the cell division site, and the actin cytoskeleton. At the molecular‐function level, downregulated sites were enriched for cytoskeletal motor activity and multiple binding activities (e.g., anion, GTPase, steroid, ribonucleotide, lipid, and phospholipid binding), indicating that CHV1 infection is accompanied by reduced phosphorylation within modules linked to polarised growth, endomembrane trafficking, ER‐associated processes, and cytoskeleton‐related functions.

In contrast, the normalised upregulated phosphosites showed strong enrichment for phosphorylation‐centred regulatory processes, including mitotic cytokinesis, mRNA catabolic processes (including nuclear‐transcribed mRNA catabolism), and negative regulation of gene expression and broader metabolic processes, together with hallmark phosphorylation terms such as protein phosphorylation, phosphorylation, and protein autophosphorylation. Molecular‐function enrichment in the upregulated set was dominated by kinase‐associated activities, including kinase activity, protein kinase activity, and protein serine/threonine kinase activity, as well as phosphotransferase activity (alcohol group as acceptor), suggesting enhanced phosphorylation regulation of kinase‐driven signalling and cytokinesis‐related circuitry upon infection.

KEGG analysis further supported this divergence (Figure 2b): the downregulated phosphosites were enriched in nucleocytoplasmic transport, MAPK signalling (yeast), ATP‐dependent chromatin remodelling, proteasome‐related processes, ribosome biogenesis in eukaryotes, endocytosis, and lysosome pathways, whereas the upregulated phosphosites were associated with N‐glycan biosynthesis, proteasome, base excision repair, the mRNA surveillance pathway, aminoacyl‐tRNA biosynthesis, protein processing in the ER, RNA degradation, and ribosome. Notably, certain pathways (e.g., proteasome) were enriched in both directions, indicating that CHV1 infection does not uniformly activate or suppress entire pathways but instead drives fine‐scale rewiring through differential regulation of specific phosphorylation nodes within shared functional systems.

### 2.3 Hypovirus Infection Increases CpIre1 Phosphorylation

**FIGURE 3:**
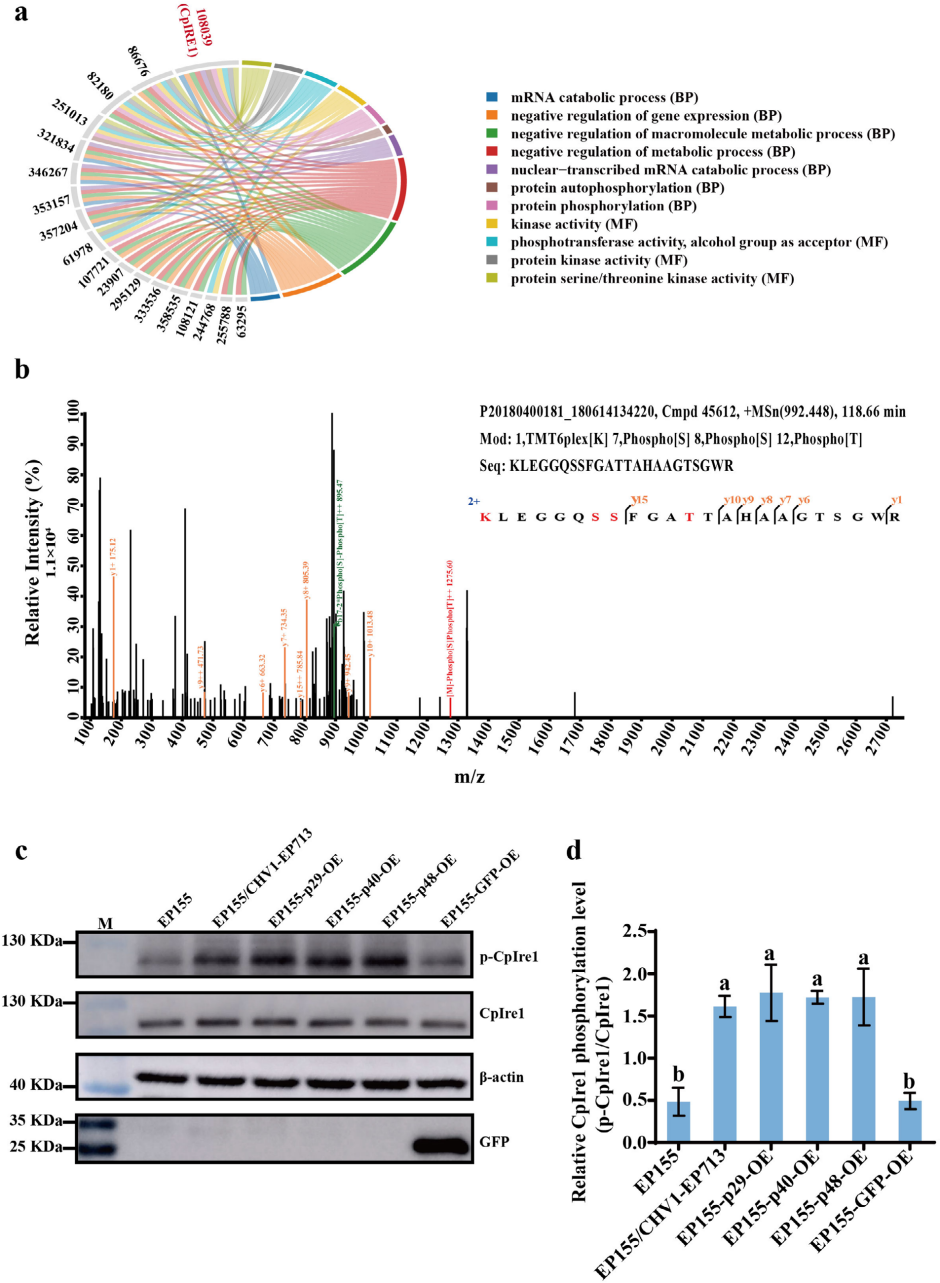
Effect of hypovirus infection on the phosphorylation level of CpIre1 protein. (a) Protein–GO chord diagram highlighting phosphorylation‐related functions. The chord diagram shows associations between selected genes (labelled by gene IDs) and enriched Gene Ontology (GO) terms in Biological Process (BP) and Molecular Function (MF) categories related to phosphorylation/kinase activities. Coloured sectors denote GO terms, and ribbons represent gene‐to‐term links. The diagram was generated using R. (b) MS/MS spectra of the detected phosphorylated peptides of CpIre1. The identified peptide sequence is shown, with red‐labelled serine (S) indicating phosphorylation sites. The detected modifications include TMT6plex labelling at lysine (K) residues and phosphorylation at specific serine (S) and threonine (T) residues. (c) Western blot analysis of CpIre1 protein expression in strains EP155, EP155/CHV1‐EP713, EP155‐p29‐OE, EP155‐p40‐OE, EP155‐p48‐OE, and EP155‐GFP‐OE using anti‐IRE1, anti‐p‐IRE1, anti‐β‐Actin, and anti‐GFP (as a loading control) antibodies. (d) Quantification of the western blot results using ImageJ, with the relative ratio of p‐CpIRE1 to CpIRE1 calculated. Error bars represent standard deviations from three independent replicates. Different letters above the bars indicate statistically significant differences between treatments (ANOVA followed by Tukey's test, *p* < 0.05).

Following enrichment analysis of differentially phosphorylated proteins, we further filtered significantly enriched phosphorylation‐related pathways and constructed a protein–GO pathway chord diagram (Figure 3a). Among the 18 identified proteins, CpIre1 (JGI Protein ID: 108039) exhibited the most notable phosphorylation change (Table S4), implying a key role for CpIre1 in the regulation of phosphorylation signalling during CHV1 infection. Therefore, CpIre1 was chosen as the primary candidate for further studies. CpIre1—a key sensor of the UPR pathway—exhibited a 2.13‐fold increase in phosphorylation in EP155/CHV1‐EP713 compared to the EP155 control (Figure 3b and Table S3). Western blot analysis using p‐IRE1 and IRE1 antibodies confirmed the correctness of the MS data (Figure 3c,d). To further investigate which viral protein is involved in the regulation of CpIre1 phosphorylation, we expressed three key viral proteins in the host fungus EP155 with recombinant plasmid pCPXG418‐p29, pCPXG418‐p40, or pCPXG418‐p48. To rule out nonspecific effects, we used GFP overexpression as a control. The results showed that all three viral proteins enhanced the phosphorylation of CpIre1, which was confirmed to be a specific effect of the viral proteins and not an artefact of protein overexpression (Figure 3c,d). Furthermore, no direct interaction was detected between p29, p40, or p48 with CpIre1 in yeast two‐hybrid assays (Figure S3), indicating the involvement of an indirect regulatory mechanism.

Moreover, to test whether the change in CpIre1 phosphorylation level is a specific response to the virus, we examined the CpIre1 phosphorylation level of EP155 under heat stress (30°C) or oxidative stress (H_2_O_2_). As shown in Figure S4, both treatments resulted in increased CpIre1 phosphorylation without a notable change in CpIre1 protein levels, indicating a general stress‐responsive phosphorylation rather than a virus‐specific effect.

### 2.4 Phosphorylation of Ser‐896 and Ser‐897 in CpIre1 Is Essential for the Development and Virulence of *C. parasitica*

CpIre1 proteins contain several essential domains, including the ER luminal domain, transmembrane domain, serine/threonine kinase domain, and ribonuclease domain, all of which are involved in sensing unfolded proteins and initiating the UPR (Mishiba et al. 2019; Zhou et al. 2006). As shown in Figure 3b, Ser‐896 and Ser‐897 of the CpIre1 were the sites of phosphorylation and located within the kinase domain of CpIre1. Sequence alignment showed that CpIre1 shares 59.2%–33.4% identity with orthologues of representative species from filamentous fungi, yeast, plant, insect, and human (Figure S5a). Of importance, Ser‐896 and Ser‐897 of the CpIre1 are conserved in all Ire1 proteins (Figure S5b), suggesting that phosphorylation of these two serines may be essential for the function of CpIre1.

**FIGURE 4:**
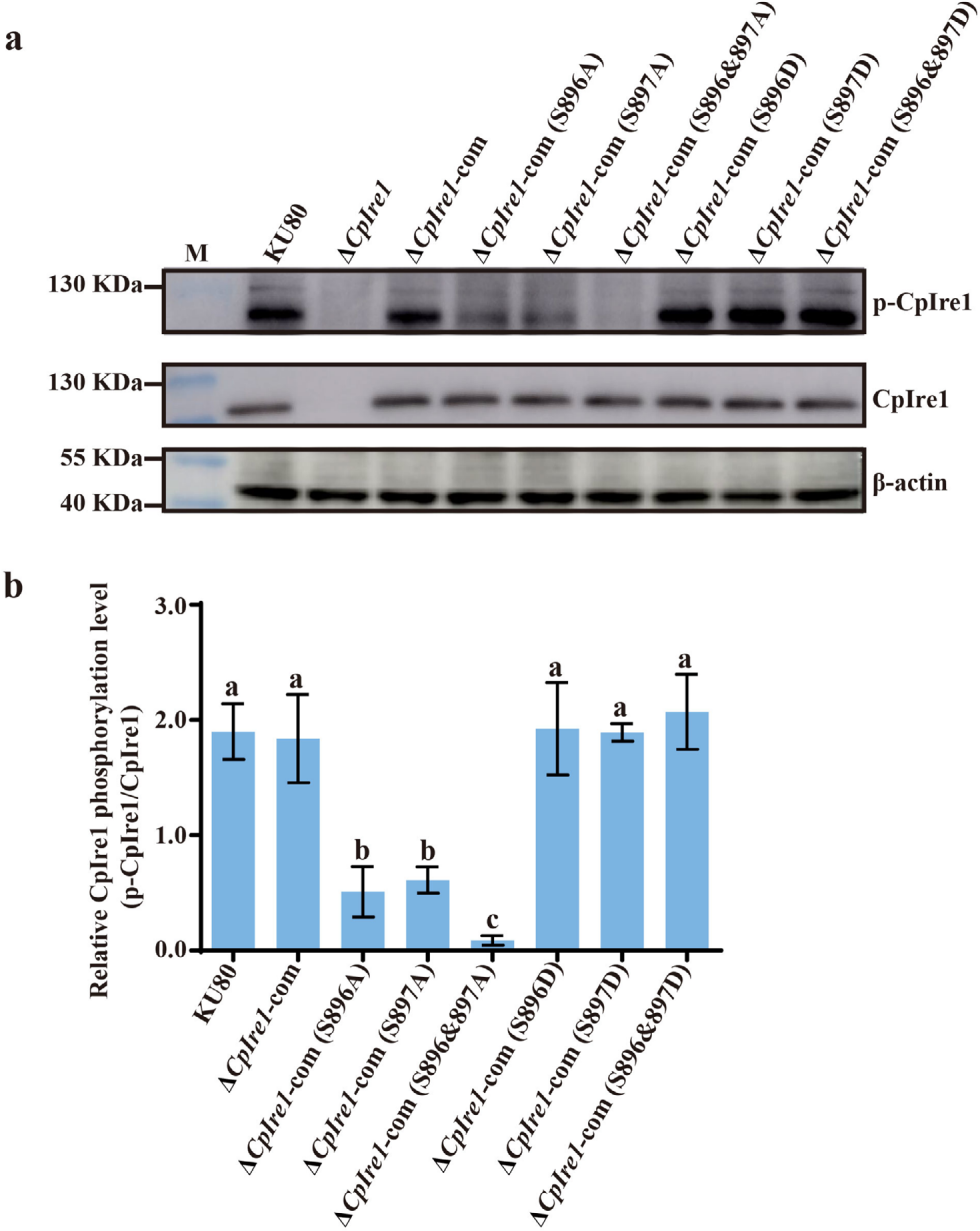
Mutation of serine residues 896 and 897 affects CpIre1 phosphorylation in *Cryphonectria parasitica*. (a) The protein expression of CpIre1 in KU80, Δ*CpIre1*, Δ*CpIre1*‐com, phospho‐deficient mutants ∆*CpIre1*‐com (S896A, S897A, and S896&897A), and phospho‐mimic mutants Δ*CpIre1*‐com (S896D, S897D, and S896&897D) was determined by western blot with an anti‐IRE1 antibody, anti‐p‐IRE1, and anti‐β‐Actin antibody (control). (b) Quantification of the western blot results using ImageJ, with the relative ratio of p‐CpIRE1 to CpIRE1 calculated. Error bars represent standard deviations from three independent replicates. Different letters above the bars indicate statistically significant differences between treatments (ANOVA followed by Tukey's test, *p* < 0.05).

To determine if phosphorylation of the two serines affects CpIre1 function, we generated a knockout mutant by replacing the target gene with the hygromycin resistance gene (*hph*) (Figure S6), followed by introduction of the complementation plasmid pCPXG418‐CpIre1 into the mutant (Figure S7). Next, we mutated these serines to alanine and aspartate to mimic the dephosphorylated and phosphorylated states as previous study, respectively (Armbruster et al. 2013). The sequencing‐confirmed *CpIre1* mutated genes were introduced into the *CpIre1* null mutant using pCPXG418‐CpIre1(S896A), pCPXG418‐CpIre1(S897A), pCPXG418‐CpIre1(S896&897A), pCPXG418‐CpIre1(S896D), pCPXG418‐CpIre1(S897D), and pCPXG418‐CpIre1(S896&897D) (Figure S7). Compared with the wild‐type and ∆*CpIre1*‐com strains, single‐site phospho‐deficient (S896A, S897A) mutants showed significantly reduced CpIre1 phosphorylation levels, while double‐site (S896&897A) alanine mutants completely abolished phosphorylation. In contrast, the phospho‐mimic mutants S896D and S897D, as well as the combined mutant S896&897D, maintained phosphorylation levels comparable to wild‐type CpIre1. Reintroduction of wild‐type *CpIre1* into the null mutant fully restored phosphorylation to normal levels (Figure 4).

**FIGURE 5:**
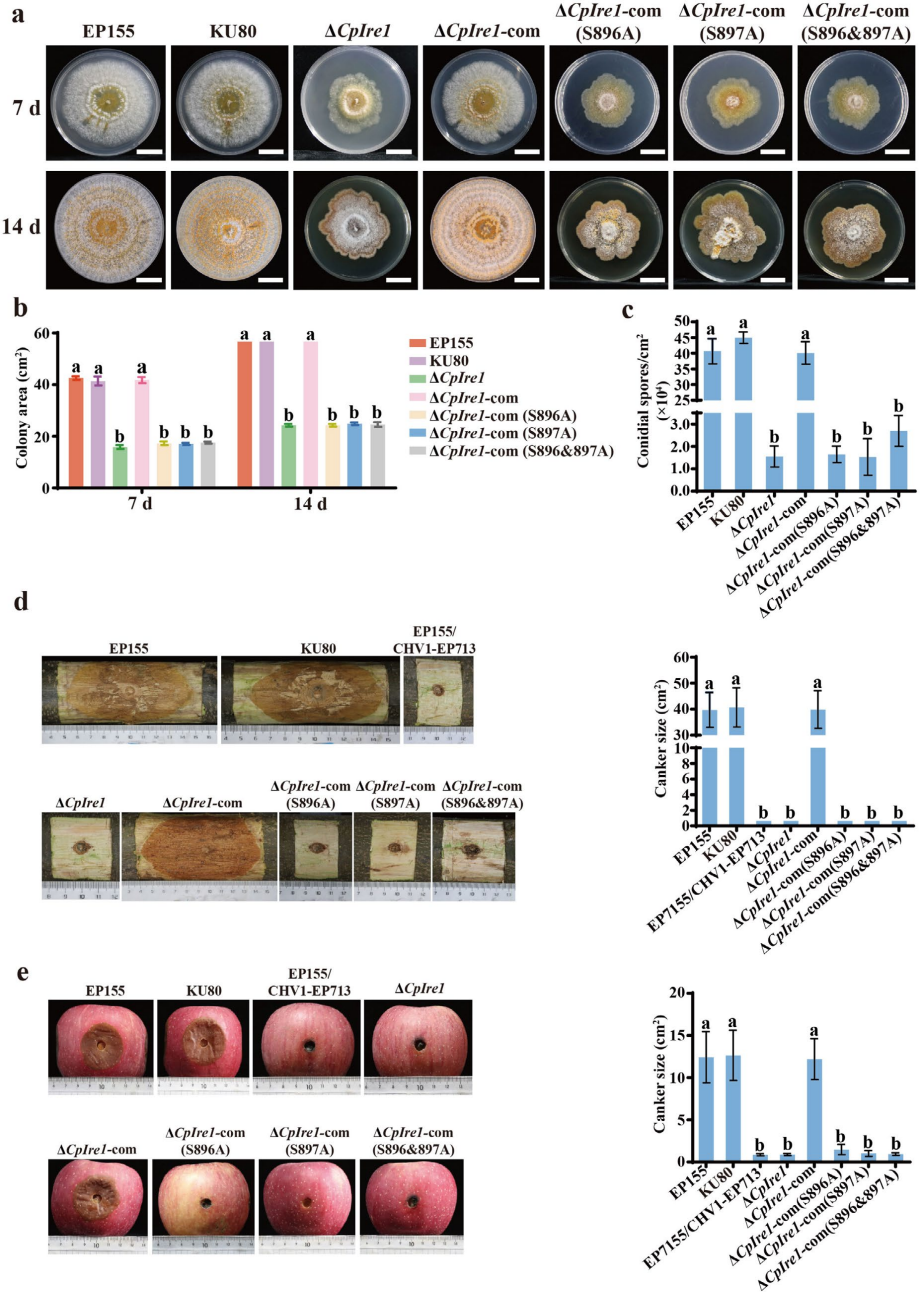
Analysis of colony morphology, sporulation, and virulence of *CpIre1* deletion mutants. (a) Colony morphology of the mutants on potato dextrose agar. Photographs were taken at 7 and 14 days after inoculation. Scale bar = 2 cm. (b) Mutant colony areas were measured at 7 and 14 days post‐inoculation (dpi). (c) Sporulation levels of the tested strains. Spores were harvested and counted at 14 dpi. (d) Dormant Chinese chestnut stems were inoculated with the indicated strains and kept at 26°C. Canker sizes were measured and photographed at 25 dpi. (e) Red Fuji apples were inoculated with the tested strains, maintained at 26°C, and cankers were measured and photographed at 10 dpi. Different letters above the columns indicate statistically significant differences between treatments (ANOVA followed by Tukey's test, *p* < 0.05).

Furthermore, when cultivated on potato dextrose agar (PDA) plates, the *CpIre1* deletion mutant exhibited slower growth, an irregular colony margin, and reduced sporulation compared to the wild‐type strain EP155 and the parent strain KU80 (Figure 5a–c). The deletion of *CpIre1* led to a significant decrease in virulence on both chestnut stems and apple fruits (Figure 5d,e). Moreover, the Δ*CpIre1* strains showed increased sensitivity to H_2_O_2_, SDS, NaCl, and Congo red compared to the EP155 and KU80 strains (Figure S8). Genetic complementation successfully rescued all associated phenotypic defects in the ∆*CpIre1*‐com strain. Notably, three phospho‐deficient mutants ∆*CpIre1*‐com (S896A, S897A, and S896&897A) failed to rescue the phenotypic defects of the Δ*CpIre1* strain, from growth and sporulation to pathogenicity (Figure 5). In contrast, all phospho‐mimic mutants ∆*CpIre1*‐com (S896D, S897D, and S896&897D) displayed growth, sporulation, and pathogenicity capacities indistinguishable from the wild‐type strain (Figure S9). Collectively, our findings demonstrate that CpIre1 phosphorylation at Ser‐896 and Ser‐897 plays a crucial role in regulating the development and virulence of *C. parasitica* .

### 2.5 Phosphorylation of CpIre1 Is Required for ER Stress Response in *C. parasitica*

**FIGURE 6:**
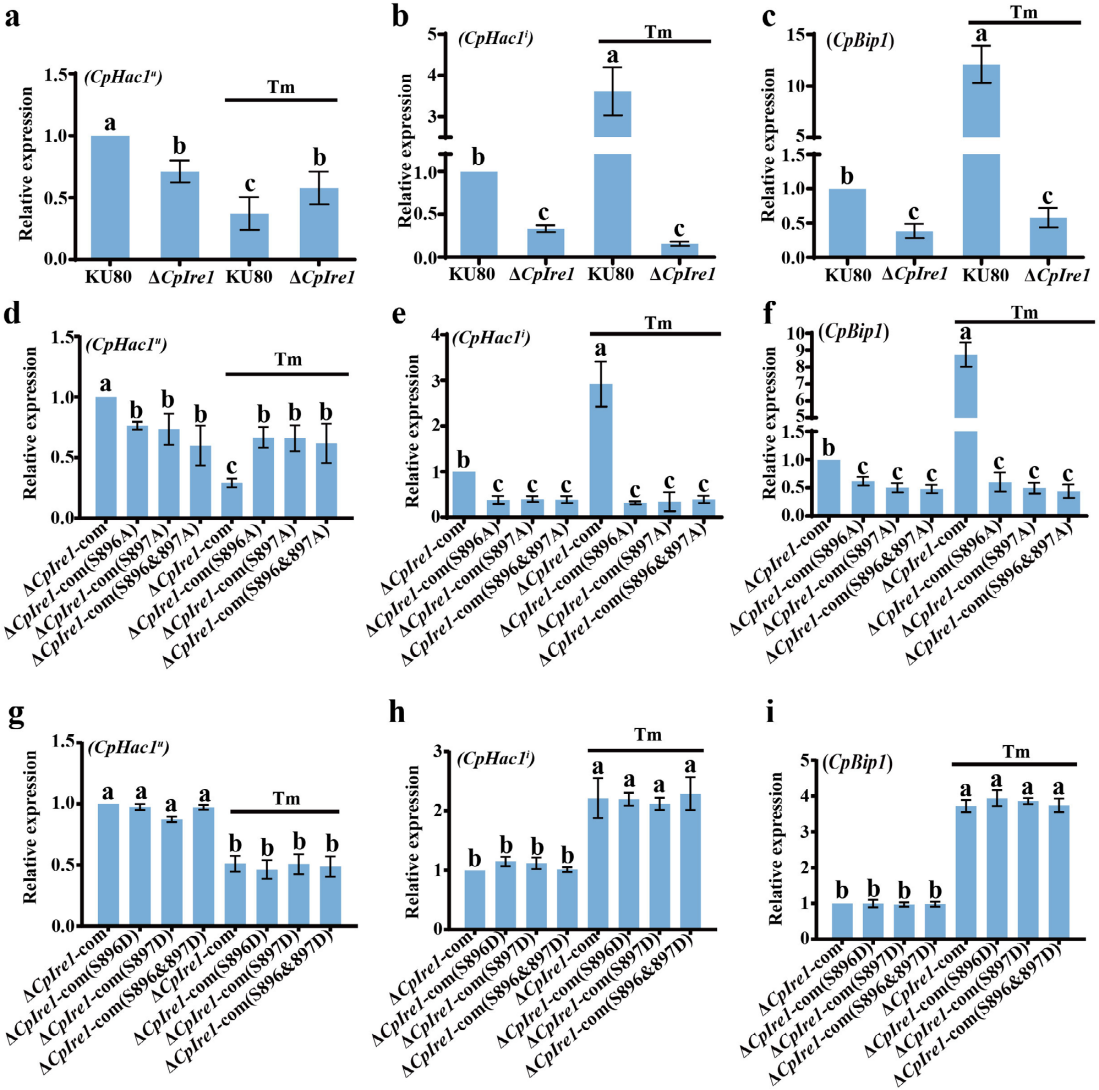
Phosphorylation of CpIre1 is essential for the endoplasmic reticulum (ER) stress response in *Cryphonectria parasitica*. (a–c) Reverse transcription‐quantitative PCR (RT‐qPCR) analysis of ER stress response genes. RNA was isolated from wild‐type (KU80) and Δ*CpIre1* strains after 7 days of incubation on potato dextrose agar (PDA) supplemented with 0.5 μM tunicamycin (Tm). Specific primers were used to quantify the expression of unspliced *CpHac1* ^*u*^ (a), spliced *CpHac1* ^*i*^ (b), and *CpBip1* mRNA (c). (d–f) RT‐qPCR analysis of ER stress response genes. RNA was isolated from the wild‐type (KU80) strain and strains with point mutations in the phosphorylation sites. (g–i) RT‐qPCR analysis of ER stress‐responsive transcripts in the ∆*CpIre1* complementation strain (∆*CpIre1*‐com) and phospho‐mimic mutants (S896D, S897D, and S896&897D). These strains were cultured for 7 days on PDA supplemented with 0.5 μM Tm. Data represent mean expression values from three independent biological replicates, each with two technical replicates. Error bars represent the standard deviation. Bars with different letters (a, b, c) indicate significant differences (*p* < 0.05).

To verify the function of CpIre1 in ER stress response, specific primers were designed to detect the unspliced (*CpHac1* ^u^) and spliced (*CpHac1* ^i^) forms of *CpHac1* mRNA using reverse transcription‐quantitative PCR (RT‐qPCR) analysis. Under ER stress induced by 0.5 μM tunicamycin (Tm), the expression level of unspliced *CpHac1* ^*u*^ exhibited a marked decrease in the KU80 (Figure 6a), while it remained unchanged in the Δ*CpIre1* mutant. Conversely, the spliced form of *CpHac1* ^*i*^ showed a substantial increase in the Tm‐treated KU80 (Figure 6b), whereas the phenomenon was nearly completely inhibited in the Tm‐treated Δ*CpIre1* mutant. In addition, RT‐qPCR analysis also revealed a significant upregulation of the UPR target gene *CpBip1*, encoding an ER‐resident chaperone, in Tm‐treated KU80 but not Tm‐treated Δ*CpIre1* (Figure 6c). These findings demonstrate that the *CpIre1* gene is essential for the ER stress response in *C. parasitica* .

Furthermore, to examine whether phosphorylated CpIre1 is essential for mediating the ER stress response, we also performed Tm treatment on three phospho‐deficient mutants ∆*CpIre1*‐com (S896A, S897A, and S896&897A). Analysis of *CpHac1* mRNA splicing revealed that the expression level of the unspliced form *CpHac1* ^u^ was significantly decreased following Tm treatment in Δ*CpIre1*‐com, but not in three mutants (Figure 6d). The generation of the spliced form *CpHac1* ^i^ was completely abolished in three mutants compared with Δ*CpIre1*‐com (Figure 6e). Additionally, the ER chaperone gene *CpBip1* of the three mutants was not effectively activated upon Tm stimulation (Figure 6f). In contrast, the phospho‐mimic strains ∆*CpIre1*‐com displayed a typical ER‐stress transcriptional response, consistent with the Δ*CpIre1*‐com (Figure 6g–i). These findings strongly confirm that Ser‐896 and Ser‐897 are the core phosphorylation sites in CpIre1 that mediate the ER stress response. Mutations at these sites completely block the transduction of the UPR signalling pathway, indicating that CpIre1 regulates downstream stress responses through a phosphorylation‐dependent mechanism.

### 2.6 Phospho‐Deficient *CpIre1* Mutation Reduces the Accumulation of Hypovirus RNA

**FIGURE 7:**
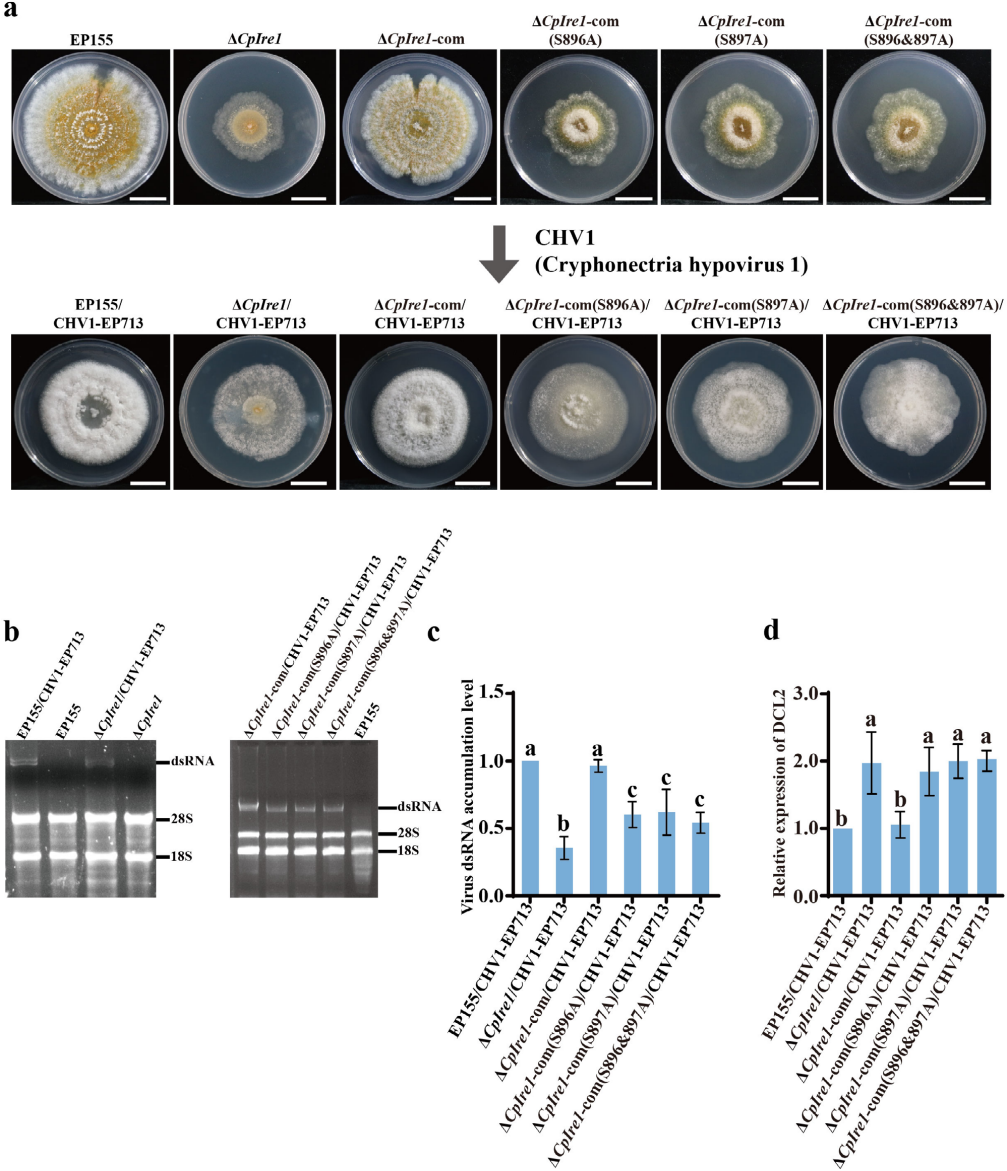
Colony morphology and viral dsRNA accumulation were examined in hypovirus‐containing *CpIre1* mutants. (a) The colony morphology of hypovirus‐free and hypovirus‐containing *CpIre1* mutants was observed on potato dextrose agar at 7 days post‐inoculation. Scale bar = 2 cm. (b) Viral dsRNA accumulation was analysed using agarose gel electrophoresis in hypovirus‐containing *CpIre1* mutants. (c) Reverse transcription‐quantitative PCR (RT‐qPCR) analysis of viral RNA levels in the hypovirus‐infected strains. (d) RT‐qPCR analysis of *DCL2* gene expression levels in the hypovirus‐infected strains. Error bars represent the standard deviation from three independent biological replicates. There are significant differences between samples indicated by different letters on the bars (ANOVA followed by Tukey's test, *p* < 0.05).

To investigate the regulatory role of CpIre1 phosphorylation in hypovirus infection, we introduced the hypovirus CHV1‐EP713 into the following strains via hyphal anastomosis: Δ*CpIre1*, Δ*CpIre1*‐com, phospho‐deficient mutants ∆*CpIre1*‐com (S896A, S897A, and S896&897A), and phospho‐mimic mutants Δ*CpIre1*‐com (S896D, S897D, and S896&897D). Following viral infection, the *CpIre1* deletion and phospho‐deficient mutants exhibited reduced colony pigmentation and enhanced growth rates (Figure 7a), whereas the phospho‐mimic mutants displayed phenotypic characteristics similar to EP155/CHV1‐EP713 (Figure S10a). The dsRNA electrophoresis analysis revealed that viral dsRNA accumulation in the Δ*CpIre1*/CHV1‐EP713 and three phospho‐deficient mutants/CHV1‐EP713 was significantly lower than in the wild‐type strain EP155/CHV1‐EP713 and the complemented strain Δ*CpIre1*‐com/CHV1‐EP713 (Figure 7b). In contrast, the phospho‐mimic mutants maintained viral dsRNA accumulation levels comparable to the Δ*CpIre1*‐com/CHV1‐EP713 (Figure S10b). These findings were further validated by RT‐qPCR, which showed that viral dsRNA accumulation in the phospho‐deficient mutants was only about 60% of that in EP155/CHV1‐EP713 (Figure 7c), while the phospho‐mimic mutants restored normal viral accumulation levels (Figure S10c). These results demonstrate that phosphorylation of CpIre1 at S896 and S897 is essential for efficient hypovirus replication and accumulation in the fungal host. Moreover, all the hypovirus‐infected mutants produced much smaller cankers on apple fruits, comparable to EP155/CHV1‐EP713 (Figure S11).

To further elucidate the mechanism underlying hypovirus accumulation in Δ*CpIre1*, mycelial samples were collected from both the KU80/CHV1‐EP713 and the Δ*CpIre1*/CHV1‐EP713 strain for RNA‐seq analysis. Hierarchical clustering of the RNA‐seq data revealed significant differences in the heat map of mRNA expression patterns between KU80/CHV1‐EP713 and Δ*CpIre1*/CHV1‐EP713 (Figure S12a). Compared to the KU80/CHV1‐EP713, the Δ*CpIre1*/CHV1‐EP713 strain exhibited 1710 differentially expressed genes (DEGs), with 970 genes upregulated and 740 downregulated (Figure S12b and Table S5). In this study, we performed GO and KEGG pathway enrichment analyses of DEGs between KU80/CHV1‐EP713 and Δ*CpIre1*/CHV1‐EP713 samples (Figure S12c,d). Our results indicate that the deletion of *CpIre1* significantly alters the host cellular environment, potentially hindering viral replication. The DEGs were mainly enriched in oxidoreductase and hydrolase activities (especially those on carbon‐nitrogen bonds), membrane functions, single‐organism metabolic processes (including secondary metabolism), ion/small molecule transport, polysaccharide metabolism, and cell wall synthesis. These findings suggest that the mutation may disrupt membrane integrity, redox balance, and polysaccharide metabolism, which might be crucial for viral replication. KEGG pathway analysis further revealed that pathways such as glutathione, glycine/serine/threonine, β‐alanine, and carotenoid biosynthesis were upregulated, while pathways like starch/sucrose, arginine/proline, and phenylalanine/tyrosine/tryptophan biosynthesis were downregulated in the Δ*CpIre1*/CHV1‐EP713 strain. The upregulation of glutathione metabolism likely reflects an enhanced antioxidant response, whereas the downregulation of amino acid pathways may hinder viral resource hijacking, which is essential for viral protein synthesis. Notably, we observed that the Dicer‐like 2 (*DCL2*) gene, a key component in the RNA interference pathway and host antiviral defence, was upregulated in the Δ*CpIre1*/CHV1‐EP713 strain (Table S5). Further confirmation by RT‐qPCR analysis showed a significant increase in *DCL2* gene expression in the Δ*CpIre1*/CHV1‐EP713 strain and three phospho‐deficient/CHV1‐EP713 mutants (Figure 7d). This finding suggests that the Δ*CpIre1* mutation may also influence the host's antiviral defence mechanisms through the regulation of *DCL2* expression.

## 3 Discussion

Protein phosphorylation is one of the most ubiquitous post‐translational modifications, regulating multiple cellular processes in fungi, including metabolic cycle, cell cycle, gene transcription, sexual cooperation, hyphal growth, cell wall synthesis, stress and pathogenicity (Albataineh and Kadosh 2016; Johnson 2009; Mohl et al. 2017; Weber et al. 2012). While studies have explored phosphorylation in fungal responses to various stimuli, our research focuses on the effects of hypovirus infection on the phosphoproteomic profiles of *C. parasitica* . This study provides a detailed phosphoproteomic analysis of fungal responses to hypovirus infection, offering valuable insights into the dynamic regulation of phosphorylation under viral infection.

We identified 700 differentially phosphorylated sites in response to viral infection, including 174 upregulated and 526 downregulated sites (Table S3). These data show that CHV1 markedly modulates the fungal phosphoproteome in a modular manner: downregulated sites are enriched in cell polarity/cytoskeletal dynamics, vesicle‐mediated transport, and endomembrane/ER organisation, whereas upregulated sites preferentially map to cell division/sporulation‐related structures, kinase activity, and quality‐control pathways such as ER protein processing, the proteasome, and RNA surveillance/decay. Together, this pattern suggests targeted rewiring of host regulatory networks rather than random phosphorylation changes. The downregulated set aligns with the membrane‐associated replication strategy of CHV1, which induces membranous vesicles that cofractionate with viral RNA/replication activities (Jacob‐Wilk et al. 2006) and exploits trans‐Golgi network (TGN) secretory vesicles for replication (Kazmierczak et al. 2012). Enrichment of the MAPK pathway is also consistent with reports that CHV1 modulates the host MAPK cascade and infection‐associated phenotypes (Chun et al. 2019). By contrast, enrichment of ER protein processing, the proteasome, and RNA surveillance among upregulated events suggests that cellular quality‐control systems are strengthened and may also be co‐opted during infection, consistent with UPR–ERAD/proteasome crosstalk (Hwang and Qi 2018) and nonsense‐mediated decay (NMD)‐mediated shaping of UPR dynamics (Karam et al. 2015). More broadly, viruses are known to exploit or fine‐tune UPR signalling to balance replication with host cell viability (Chen et al. 2025).

Viral infections have been shown to modulate Ire1 phosphorylation and the UPR across diverse organisms. Viruses often exploit UPR signalling to enhance their replication, persistence, and interactions with the host (Zhang et al. 2024). For instance, hepatitis C virus (HCV) hijacks the UPR by altering Ire1 phosphorylation, thereby promoting viral replication and persistence in mammalian cells (Tardif et al. 2004). Similarly, Zika virus (ZIKV) infection activates both the IRE1‐XBP1 and ATF6 branches of the UPR in neural cells, contributing to viral pathogenesis and neurodevelopmental disorders (Tan et al. 2018). In plants, rice stripe virus (RSV) manipulates the UPR to facilitate infection and balance viral spread with host viability (Li et al. 2021). These reports highlight the evolutionary conservation of viral strategies to modulate UPR signalling (Chan 2014). Our study further extends this concept, showing that viral infections in fungi also modulate the UPR signalling pathway. Specifically, we show that CpIre1, a key regulator of the UPR in *C. parasitica* , undergoes phosphorylation at Ser‐896 and Ser‐897 under hypovirus infection. This phosphorylation triggers UPR activation, demonstrating that mycoviruses hijack this conserved pathway—mirroring mechanisms in animals and plants—to regulate fungal stress adaptation and virulence. The deletion of the *CpIre1* gene in *C. parasitica* resulted in significant phenotypic and molecular alterations, demonstrating its crucial role in fungal growth, virulence, sporulation, and stress adaptation. Notably, phospho‐deficient mutants exhibited similar defects, including impaired growth, reduced sporulation, and attenuated virulence. Further analysis suggested that these growth and sporulation deficiencies may stem from disrupted protein synthesis and metabolic imbalance, highlighting the essential role of *CpIre1* phosphorylation in its regulatory function. Moreover, these findings are consistent with previous studies on Ire1 orthologs in pathogenic fungi such as *M. oryzae* and *Ustilago maydis* (Heimel et al. 2013; Yang et al. 2024), indicating an evolutionarily conserved, phosphorylation‐dependent regulatory mechanism of Ire1 in fungi.

The critical role of CpIre1 in the ER stress response was validated using Tm treatment, an inhibitor of N‐linked glycosylation. In wild‐type *C. parasitica* , Tm treatment induced the canonical UPR activation marker‐*Hac1* mRNA splicing. The spliced *CpHac1* mRNA encodes a transcriptional activator that promotes the transcription of the chaperone protein‐encoding gene *CpBip1*. However, these phenomena were completely abolished in Δ*CpIre1* mutants (Figure 6). This genetic evidence demonstrates that CpIre1 is indispensable for *CpHac1* mRNA processing and subsequent UPR activation, consistent with the conserved functions of Ire1 homologues across diverse eukaryotic species (Cheon et al. 2011; Heimel et al. 2013; Miyazaki et al. 2013; Richie et al. 2009). Notably, phospho‐deficient mutants exhibited identical defects in *CpHac1* splicing under ER stress conditions, confirming that kinase activity is essential for CpIre1's sensor‐transducer function. This conclusion was further supported by impaired upregulation of the UPR target gene *Bip1* in both Δ*CpIre1* and phospho‐deficient mutants. Given that *Bip1* encodes a crucial ER chaperone involved in maintaining protein folding homeostasis, its transcriptional dysregulation directly links CpIre1 phosphorylation status to ER homeostasis regulatory capacity. The identical phenotypic defects observed in both gene deletion mutants and phosphorylation‐inactive mutants, combined with conserved molecular signatures (*Hac1* splicing/*Bip1* induction), collectively demonstrate that the phosphorylation‐dependent Ire1 activation mechanism represents an evolutionarily conserved regulatory paradigm that plays a vital role in maintaining ER functional integrity in eukaryotes.

The accumulation level of CHV1 virus was significantly decreased in Δ*CpIre1* and phospho‐deficient mutants (Figure 7b,c), highlighting the critical role of Ire1‐mediated UPR in viral replication. By RNA‐seq analysis, we revealed that some pathways important for viral replication were significantly downregulated in ∆*CpIre1*/CHV1‐EP713, consistent with some previous studies. This mechanism is evolutionarily conserved: In the context of flavivirus infection, the IRE1 pathway supports viral replication through a dual regulatory mechanism: (i) enhancing host cell protein biosynthesis, and (ii) promoting lipid biogenesis to provide essential building blocks for viral replication. This mechanistic insight was experimentally validated in tick‐borne flavivirus infection of astrocytes, where IRE1‐specific inhibitor treatment resulted in a significant reduction of viral replication efficiency (Breitkopf et al. 2021). More significantly, during transmissible gastroenteritis virus (TGEV) infection, IRE1α‐mediated UPR orchestrates a sophisticated gene regulatory network to facilitate viral replication (Ma et al. 2018). Collectively, these cross‐kingdom examples suggest that manipulation of UPR pathways represents a pivotal strategy for viruses to alleviate ER stress and maintain functional replication factories.

Previous studies have shown that Ire1, in addition to splicing Hac1 during the UPR, can also regulate Ire1‐dependent decay (RIDD) via its RNase activity, selectively cleaving and promoting the degradation of a defined set of host transcripts, thereby modulating cellular RNA homeostasis (Moore and Hollien 2015; Le Thomas et al. 2021). Furthermore, accumulating evidence suggests that viruses often exploit or fine‐tune the UPR signalling pathway to balance enhanced replication with the preservation of host cell viability (Chen et al. 2025). In a Japanese encephalitis virus (JEV) model, the infection‐induced UPR activates RIDD, and inhibiting IRE1 RNase activity reduces viral protein levels and viral titres (Bhattacharyya et al. 2014), suggesting a pro‐viral role for the IRE1–RIDD axis. Consistent with these findings, our study reveals that deletion of CpIre1 during CHV1 infection leads to significant upregulation of 970 genes (Table S5). Notably, among these upregulated genes, we found various transcripts that correspond to reported features of RIDD targets, including those involved in the secretory/membrane system and ER processing (Moore and Hollien 2015), suggesting that loss of CpIre1 impairs Ire1‐mediated RNA cleavage and degradation process, resulting in transcript accumulation. Especially, knockout of the *CpIre1* gene caused a notable increase in *DCL2* transcript levels, which is known to be a primary component of the antiviral pathway (Segers et al. 2007). Based on this observation, we speculate that CpIre1 deficiency abolishes RIDD‐like activity, thereby alleviating (directly or indirectly) the repression of DCL2 and leading to increased DCL2 expression. Elevated DCL2 levels may subsequently enhance the processing and clearance of viral RNA, ultimately suppressing viral accumulation. Due to the limited information on the Ire1‐mediated UPR pathway in fungal viruses, further studies are required to clarify the detailed regulatory mechanism.

**FIGURE 8:**
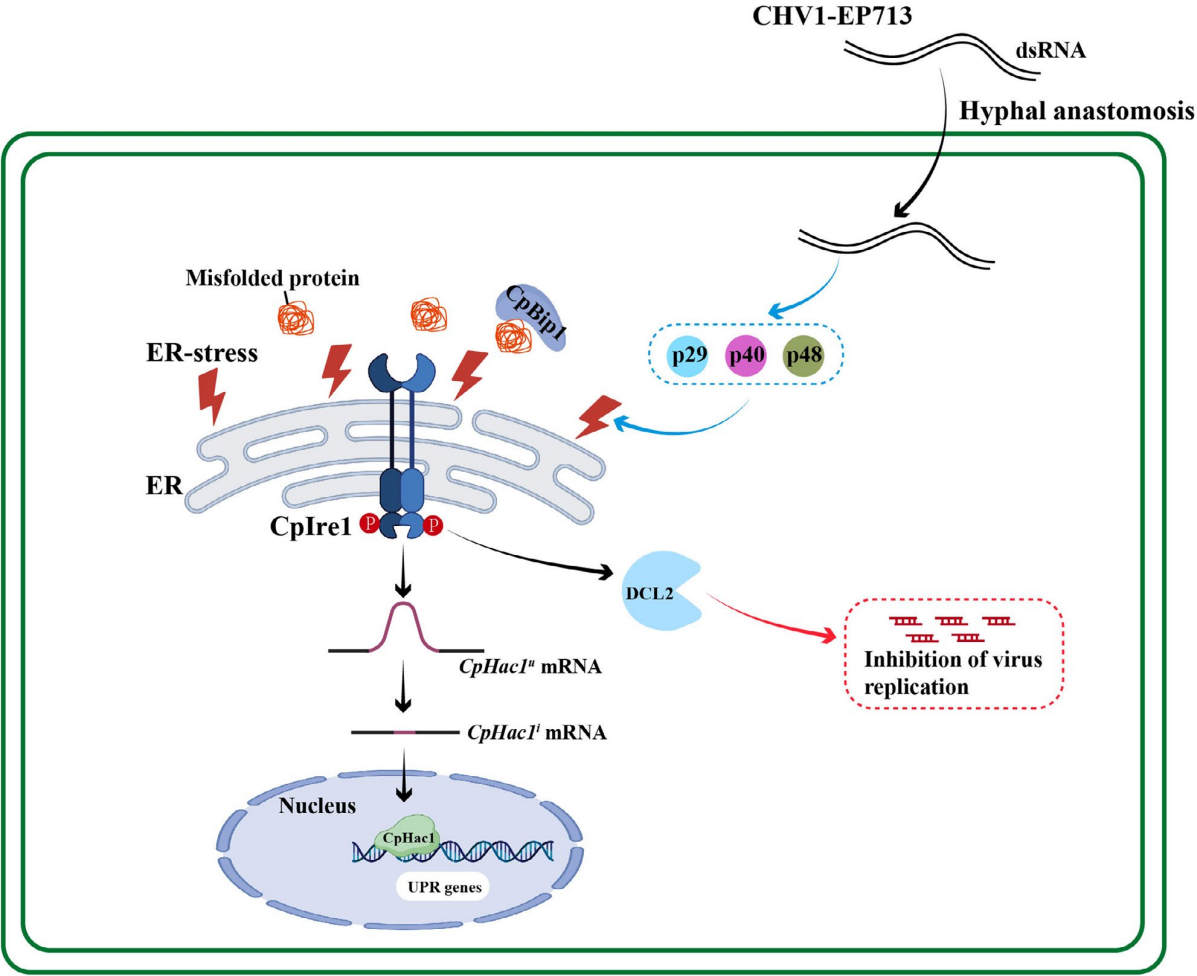
Hypovirus‐induced phosphorylation of CpIre1 modulates the endoplasmic reticulum (ER) stress response in *Cryphonectria parasitica*.

In conclusion, this study highlights the critical role of phosphorylation in fungal development, UPR activation, stress adaptation, and viral replication in *C. parasitica* . We demonstrate that CpIre1‐mediated ER homeostasis, fungal pathogenicity, and hypovirus RNA accumulation require phosphorylation at conserved Ser‐896 and Ser‐897 residues. Our research enhances our understanding of how hypovirus influences Ire1‐mediated UPR signalling in fungi. The schematic model shown in Figure 8 summarises our findings.

## 4 Experimental Procedures

### 4.1 Fungal Strains, Culture Conditions and Phenotype Analysis

*Cryphonectria parasitica* strains were used in the present study: the wild‐type (WT) strain EP155, its isogenic strain EP155/CHV1‐EP713 (synthetic hypovirus CHV1‐EP713 infected EP155) (Chen et al. 1994), the highly efficient homologous recombination strain KU80 (a mutant of EP155 disrupted in the KU80 gene) and derived mutant strains (Lan et al. 2008). Fungal cultures were grown at 26°C on PDA under a 12 h light/dark cycle to facilitate phenotypic observations and the extraction of DNA and RNA (Hillman et al. 1990). EP complete liquid medium was used for protein preparation as previously described (Puhalla and Anagnostakis 1971). Growth rate, pigmentation, and conidiation changes of *C. parasitica* were measured using methods as previously described (Kim et al. 1995). Stress response was evaluated by measuring fungal growth on PDA supplemented with H_2_O_2_, SDS, NaCl, and Congo red as appropriate. At least three biological replicates were analysed for all experiments.

### 4.2 Protein Extraction, Trypsin Digestion and TMT Labelling

Fungal proteins were extracted according to the protocol modified from Wang et al. (2014). Protein concentrations were determined by the BCA protein assay and stored at −80°C until use. The quantified samples were then digested with trypsin as described with some minor modifications (Wiśniewski et al. 2009). Briefly, 400 μg of protein for each biological replicate was incubated with 100 mM dithiothreitol (DTT) at 100°C for 5 min, then cooled to room temperature. The detergent, DTT and other low‐molecular‐weight components were removed by repeated ultrafiltration (Millipore, 10 kDa) using 200 μL UA buffer (8 M urea, 150 mM Tris–HCl pH 8.0). Then, 100 μL iodoacetamide (50 mM IAA in UA buffer) was added and the samples were incubated for 30 min in darkness. The filters were washed with 100 μL UA buffer three times and then with 100 μL NH_4_HCO_3_ buffer twice. Finally, the protein suspensions were digested with 8 μg trypsin (Promega) in 40 μL NH_4_HCO_3_ buffer at 37°C for 16–18 h, and the resulting peptides were collected as a filtrate. The peptide concentration was quantified using UV light spectral density at 280 nm. Each peptide sample derived from an independent biological replicate was labelled separately with distinct TMT reagents (TMTsixplex, Thermo Fisher Scientific) following the manufacturer's protocol (e.g., EP155 replicates labelled with TMT‐126, ‐127, ‐128 and EP155/CHV1‐EP713 replicates labelled with TMT‐129, ‐130, ‐131). After labelling, all six TMT‐labelled peptide samples were combined into one pooled mixture for subsequent high‐pH reversed‐phase fractionation and LC–MS/MS analysis. Peptide spectra were assigned to their biological sources based on reporter ion intensities corresponding to each TMT channel.

### 4.3 Affinity Enrichment of Phosphorylated Peptides

The labelled mixed peptides were dried by vacuum centrifugation, and phosphopeptide enrichment was subsequently performed using the High‐Select Fe‐NTA Phosphopeptides Enrichment kit (Thermo Scientific), then freeze‐dried under vacuum, and dissolved by 25 μL 0.1% formic acid for mass spectrometry as described previously (Ferguson et al. 2020).

### 4.4 LC–MS/MS Data Analysis

The enriched peptides were separated using a nanolitre flow rate HPLC liquid phase system EASY‐nLC1000 (Thermo Finnigan). For liquid phases A and B, a 0.1% formic acid‐water solution and 0.1% formic acid‐acetonitrile solution were used, respectively. The column was balanced with 95% liquid A. The sample was loaded from the autosampler to the Thermo Scientific EASY column (2 cm × 100 μm 5 μm‐C18), and then separated by analysis column (75 μm × 250 mm 3 μm‐C18) with a flow rate of 250 nL/min. The relevant liquid phase gradients were set as follows: 0–2 min, there is a linear gradient of 2%–5% in liquid B; 2–122 min, there is a linear gradient of 5%–24% in liquid B; 122–227 min, there is a linear gradient of 24%–40% in liquid B; 227–232 min, there is a linear gradient of 40%–100% in liquid B; 232–242 min, the liquid B is maintained at 100%. Separated samples were further analysed by the Q‐Exactive mass spectrometer (Thermo Finnigan) with a mass range of 300–1800 *m*/*z* in the positive ion mode for 240 min. The resolution of secondary mass spectrometry was 17,500 at *m*/*z* 200.

### 4.5 Protein Identification and Quantitative Analysis

Data from mass spectrometry analysis was stored in RAW files. To identify and quantify the protein samples, the MS raw data were searched with the Proteome Discoverer v. 1.4 and Mascot v. 2.2 against the proteome database deduced from the *C. parasitica* genome (Wiśniewski et al. 2009). Relevant parameters were set as follows: enzyme = trypsin; max missed cleavages = 2; peptide mass tolerance = ±20 ppm; fragment mass tolerance = 0.1 Da; fixed modifications = carbamidomethyl (C), TMT 6plex (N‐term), TMT 6plex (K); variable modifications = oxidation (M), phospho (ST), phospho (Y). Phosphorylated peptides were analysed using Proteome Discoverer v. 1.4. The score threshold for peptide identification was set at a 1% false discovery rate (FDR) and phosphoRS site probability > 0.75. TMT reporter ion intensities were normalised to the total signal within each channel (representing total protein amount) to correct for loading and labelling variations, as previously outlined (Gao et al. 2021). Differential abundance was considered significant when the fold change was > 1.5 (upregulated) or < 0.6 (downregulated) with a *p* ≤ 0.05.

### 4.6 Western Blot

Fungal proteins were prepared and subsequently separated with 12% SDS‐PAGE, and then transferred to a polyvinylidene fluoride (PVDF) membrane (Millipore). The membranes were blocked with 5% non‐fat milk and incubated with primary antibodies (anti‐β‐actin, 1:5000), anti‐phospho‐IRE1 (Abcam, clone EPR5253, cat. #ab124945, 1:500), anti‐IRE1 (CST, clone 14C10, cat. #3294, 1:1000), anti‐GFP (Beyotime, AG281) for 12 h at 4°C. After being washed with Tris‐buffered saline with Tween 20 (TBST), the membranes were incubated with horseradish peroxidase‐conjugated secondary antibody (1:8000 in 5% nonfat milk) for 1 h at 25°C, and the bands were detected using ECL detection reagent (Coolaber). The ECL chemiluminescence was visualised and captured by LAS 600 Imaging System.

### 4.7 Construction and Verification of Fungal Mutants

To generate *CpIre1* gene deletion mutant, KU80 strain and homologous recombination method were used as described previously (Lan et al. 2008). First, a 1003 bp fragment upstream of *CpIre1* gene was amplified with *CpIre1*‐left‐F/*CpIre1*‐left‐R primers. Next, a 1000 bp fragment downstream of *CpIre1* gene was amplified with *CpIre1‐*right‐F/*CpIre1‐*right‐R primers. Primers hph‐F/hph‐R were used to amplify the hygromycin resistance (*hph*) gene fragment. By ligating the three amplified products, a fusion PCR product was obtained. This product was then transformed into the protoplasts of KU80. The Δ*CpIre1* transformants were selected on PDA supplemented with hygromycin (40 μg/mL), and then confirmed using PCR (*CpIre1*‐all‐F/*CpIre1*‐all‐R) and Southern blotting according to the method described in Sambrook and Russell (2001). Subsequently, the *CpIre1* deletion mutants were purified to nuclear homogeneity by single‐spore isolation.

For complementation, the *CpIre1* ORF with its native promoter was amplified using primer pairs (*CpIre1*‐ORF‐F/*CpIre1*‐ORF‐R). Subsequently, it was inserted into the transformation vector pCPXG418 containing the G418 resistance gene (Chen et al. 2011). The recombinant plasmid pCPXG418‐*CpIre1* was transformed into protoplasts of the *CpIre1* deletion mutant. Hygromycin (40 μg/mL) and G418 (25 μg/mL) were included in the medium for selection of transformants. Primers G418‐CpIre1‐F/G418‐CpIre1‐R were used to confirm the complementation strain Δ*CpIre1*‐com.

For site‐directed mutagenesis, point mutations of CpIre1 (S896A, S897A, S896&897A, S896D, S897D, S896&897D) were introduced using the Mut Express II rapid mutagenesis kit V2 (Vazyme Biotech) with pCPXG418‐*CpIre1* as the template. Each mutant plasmid was confirmed by DNA sequencing and then transformed into the Δ*CpIre1* strain. All the primers used in this study are listed in Table S6.

### 4.8 Hypovirus Transmission

Virus transmission was performed through hyphal anastomosis by the coculture of virus‐containing strain EP155/CHV1‐EP713 and recipient fungal strains on a PDA plate, as described previously (Cortesi et al. 2001). The presence of hypovirus was confirmed by the isolation of dsRNA from single‐spored strains.

### 4.9 RNA Isolation and RT‐qPCR

Fungal hyphae cultured for 7 days were collected and then frozen with liquid nitrogen. Total RNA was extracted from fungal hyphae using MiniBEST Plant RNA Extraction Kit (Takara). The purified RNA was reverse transcribed into cDNA using HiScript III RT SuperMix (Vazyme Biotech). With 18S rRNA as the internal reference, the fluorescence was quantified using Takara SYBR dye and a LightCycler 480 II quantitative PCR instrument from Roche. The relative *CpIre1* gene expression was calculated using the 2^−ΔΔ*C*t^ method as described previously. The primer sequences used are listed in Table S6.

The relative accumulation levels of viral dsRNA were determined by quantitative real‐time PCR as described previously by Lin et al. (2007). cDNAs specific for viral plus‐ and minus‐strand RNA were generated with primers RTQ2 and RTQ1, respectively, using a HiScript III RT SuperMix (Vazyme Biotech). Each cDNA synthesis reaction mixture contained primer RT‐18S‐F/RT‐18S‐R, which was complementary to the portion of *C. parasitica* 18S rRNA. The primer sets used for real‐time quantitative PCR (qPCR) were as follows: RTQ1 and RTQ2 for viral RNA, and RT‐18S‐R/F for 18S rRNA. The viral cDNA levels were normalised to the cDNA levels of 18S rRNA for quantification.

### 4.10 Transcriptome Sequencing and Analysis

The wild‐type and mutant strains were cultured on PDA at 26°C, and mycelia were collected at 7 days post‐inoculation (dpi). RNA extraction, sequencing and data analysis were carried out by Genedenovo company (Guangzhou, China). Three independent biological replicates were used for each sample. A sequencing library was constructed and then sequenced on the Illumina HiSeq 4000 platform (Illumina). High‐quality clean reads were obtained by using Fastp software (v. 0.18.0) to filter out reads with adapters, over 10% unknown nucleotides, and low‐quality reads (*q* ≤ 20) (Liu et al. 2020). With HISAT2 v. 2.4 software (Kim et al. 2019), clean data from each sample were mapped to the genome of *C. parasitica* (https://genome.jgi.doe.gov/portal/Crypa2/Crypa2.download.ftp.html). DEGs were analysed using DESeq2 (Love et al. 2014). DEGs were considered significant if their log_2_(FC) > |1| and their FDR < 0.05.

### 4.11 Virulence Assays

The virulence of fungal strains was detected using dormant stems of Chinese chestnut ( *Castanea mollissima* ) and Red Fuji apple with triplicate per fungal strain as previously described (Shi et al. 2014). Stems or apples inoculated with *C. parasitica* were incubated at 26°C, and the sizes of canker lesions were measured at 25 dpi or 10 d, respectively.

### 4.12 Bioinformatics Analysis

The protein function was annotated by Blast2GO including BLAST, mapping, annotation and annotation augmentation (Hulsegge et al. 2009). The KAAS (KEGG Automatic Annotation Server) software was used to perform KEGG pathway annotation on the target protein collection (Moriya et al. 2007). Fisher's Exact Test was used to compare the distribution of each GO classification or KEGG pathway in the target protein set and the overall protein set, and to analyse the enrichment of GO annotations or KEGG pathway annotations on the target protein set. The motif‐X software was used to analyse the motif of sequences with amino acids in specific positions of modifier‐13‐mers (six amino acids upstream and downstream of the site) in all protein sequences (Chou and Schwartz 2011).

## Author Contributions

Lijiu Zhao: methodology, software, data curation, formal analysis, writing – original draft. Feng Wang and Fengyue Chen: validation and formal analysis. Suzhen Su: methodology. Jinfeng Qiu: methodology. Baoshan Chen and Ru Li: funding acquisition, writing – review and editing, and supervision. All authors have read and agreed to the published version of the manuscript.

## Funding

This work was supported by the Foundation for Innovative Research Groups of the National Natural Science Foundation of China (32160623; 31760498).

## Conflicts of Interest

The authors declare no conflicts of interest.

## Supporting information


**Figure S1:** Mass error distribution of phosphorylated peptides. The *x*‐axis represents the mass error in parts per million (ppm), and the *y*‐axis shows the corresponding peptide scores. Each point indicates a phosphorylated peptide, with a dense cluster around zero mass error, reflecting high accuracy in peptide mass measurements.

**Figure S2:** Number of identified peptides in each conserved motif.

**Figure S3:** The yeast two‐hybrid (Y2H) assay revealed no interaction between p29, p40, or p48 and CpIre1, respectively.

**Figure S4:** Heat and oxidative stress increase CpIre1 phosphorylation without altering total CpIre1 abundance. (a) Immunoblot analysis of phosphorylated CpIre1 (p‐CpIre1) and total CpIre1 in EP155 under control temperature (26°C), heat stress (30°C), or oxidative stress (1 mM H_2_O_2_). GAPDH was used as a loading control. (b) Quantification of CpIre1 phosphorylation level expressed as the p‐CpIre1/CpIre1 ratio. Data are presented as mean ± SD (*n* = 3). Different letters indicate statistically significant differences (*p* < 0.05).

**Figure S5:** Phylogenetic analysis and sequence similarity comparison of CpIre1 homologues across different species. (a) Phylogenetic tree of CpIre1 from various organisms generated with MEGAX software. Sequence similarity between CpIre1 and other homologous proteins was assessed using DNAMAN software to compare homologous protein sequences. (b) Sequence alignment of CpIre1 and its orthologs was conducted using CLC Genomics Workbench, with red boxes indicating conserved regions in CpIre1. The box indicates conserved amino acids.

**Figure S6:** Generation of *CpIre1* gene knockout strains. (a) Schematic representation of the *CpIre1* homologous recombination construct. Lane 1: left arm of the *CpIre1* gene; Lane 2: right arm; Lane 3: *hph* gene; Lane 4: fusion fragment containing the left arm, *hph*, and right arm. (b) PCR confirmation of *CpIre1* gene knockout in mutant strains. (c) Schematic diagram of the *CpIre1* gene deletion strategy. Probe A, targeting the *hph* gene, and Probe B, targeting the right arm, were used to distinguish wild‐type and Δ*CpIre1* mutant strains based on fragment size in Southern blotting analysis. Scale bar = 1 kb. (d) Southern blotting analysis of Δ*CpIre1* mutants, using Probe A (left) and Probe B (right). Genomic DNA from fungal strains was digested with *Bsp1407* I, separated by agarose gel electrophoresis, and hybridised with Probe A and Probe B, respectively.

**Figure S7:** Construction and validation of the complementation strain and point mutation strains of *CpIre1*. (a) Diagram outlining the construction of the *CpIre1* gene complementation plasmid, pCPXG418‐com‐*CpIre1*. (b) Validation of the pCPXG418‐com‐*CpIre1* plasmid by *EcoR* I restriction enzyme digestion. Lane 1: *CpIre1* gene. Lane 2: pCPXG418 plasmid digested with *EcoR* I. Lane 3: pCPXG418‐com‐*CpIre1* plasmid. Lane 4: pCPXG418‐com‐*CpIre1* plasmid digested with *EcoR* I. (c) PCR confirmation of Δ*CpIre1*‐com. (d) Southern blotting analysis of Δ*CpIre1*‐com, using Probe B (right) (Figure S6c). (e) DNA sequencing results confirmed the construction of the point mutation plasmids.

**Figure S8:** Growth of fungal strains on PDA medium supplemented with H_2_O_2_, SDS, NaCl, or Congo red. (a) Colony morphology of wild‐type, deletion mutant, complementation mutant, and overexpression mutant strains after 7 days of incubation at 26°C. Scale bar = 2 cm. (b) Growth inhibition rate of the strains in response to stress conditions, with colony diameter on standard PDA set as 100%. All measurements were taken after 7 days at 26°C and performed in triplicate. Error bars represent the standard deviation from three independent biological replicates. There are significant differences between samples indicated by different letters on the bars (ANOVA followed by Tukey's test, *p* < 0.05).

**Figure S9:** Characterisation of colony morphology, sporulation, and virulence in phospho‐mimic *CpIre1* mutant strains. (a) Colony morphology of the mutants on PDA medium. Photographs were taken at 7 and 14 days after inoculation. Scale bar = 2 cm. (b) Mutant colony areas were measured at Day 7 and 14 post‐inoculation, respectively. (c) Sporulation levels of the tested strains. Spores were harvested and counted on Day 14 post‐inoculation. (d) Red Fuji apples were inoculated with the tested strains, maintained at 26°C, and were measured and photographed on Day 10 post‐inoculation. Letters above the columns indicate statistical significance of the difference between three treatments (ANOVA followed by Tukey's test, *p* < 0.05).

**Figure S10:** Colony morphology and viral dsRNA accumulation were examined in hypovirus‐containing *CpIre1* phospho‐mimic mutants. (a) Colony morphology of hypovirus‐free and hypovirus‐containing CpIre1 phospho‐mimic mutants grown on PDA for 7 days. Scale bar = 2 cm. (b) Viral dsRNA accumulation in hypovirus‐containing *CpIre1* phospho‐mimic mutants, as analysed by agarose gel electrophoresis. (c) Viral RNA levels in the infected strains were determined by qRT‐PCR. Error bars represent standard deviations from three independent biological replicates. Significant differences between samples (indicated by different letters) were determined by (ANOVA followed by Tukey's test *p* < 0.05).

**Figure S11:** Virulence analysis of the *CpIre1* complementation strain, phospho‐deficient mutants, and phospho‐mimic mutants infected with CHV1‐EP713. (a) Red Fuji apples were inoculated with the tested strains, maintained at 26°C, and photographed on Day 10 post‐inoculation. (b) Measurement of canker development in different fungal strains. Letters above the columns indicate statistical significance of the difference between three treatments (ANOVA followed by Tukey's test, *p* < 0.05).

**Figure S12:** RNA‐seq analysis reveals the impact of *CpIre1* deletion on gene expression in CHV1‐EP713‐infected *C. parasitica* . (a) Heatmap displaying the relative gene expression levels in Δ*CpIre1*/CHV1‐EP713 compared to the KU80/CHV1‐EP713 strain, based on RNA‐seq data. (b) Volcano plot illustrating DEGs between KU80/CHV1‐EP713 and Δ*CpIre1*/CHV1‐EP713. mRNAs with log_2_FC > 1 in Δ*CpIre1* relative to KU80 (*p* < 0.01, two‐tailed *t*‐test) are marked in red, while mRNAs with log_2_FC < −1 (*p* < 0.01, two‐tailed *t*‐test) are highlighted in blue. (c) GO enrichment analysis of DEGs, categorised by biological process (BP), cellular component (CC), and molecular function (MF). (d) KEGG pathway enrichment analysis of the DEGs. DEG refers to differentially expressed gene.

**Table S1:** Summary of identified phosphorylation sites.

**Table S2:** Analysis of the phosphorylation‐modified peptide motif.

**Table S3:** Normalisation of phosphorylation to the protein changes.

**Table S4:** Proteins identified in the protein–GO pathway chord diagram.

**Table S5:** Differentially expressed genes in KU80/CHV1‐EP713 and Δ*CpIre1*/CHV1‐EP713.

**Table S6:** Primers used in this study.

## Data Availability

The data that support the findings of this study are available from the corresponding author upon reasonable request.
