# Supplementary material for: Hypovirus‐Induced Phosphorylation of CpIre1 Modulates Unfolded Protein Response and Virulence in Cryphonectria parasitica
Source: Mol Plant Pathol. 2026 Feb 15;27(2):e70227. doi: 10.1111/mpp.70227 (PMC12907514; doi:10.1111/mpp.70227)
Supplement: Supplementary file 1 — Figure S1: Mass error distribution of phosphorylated peptides. The x‐axis represents the mass error in parts per million (ppm), and the y‐axis shows the corresponding peptide scores. Each point indicates a phosphorylated peptide, with a dense cluster around zero mass error, reflecting high accuracy in peptide mass measurements. [file MPP-27-e70227-s003.docx]

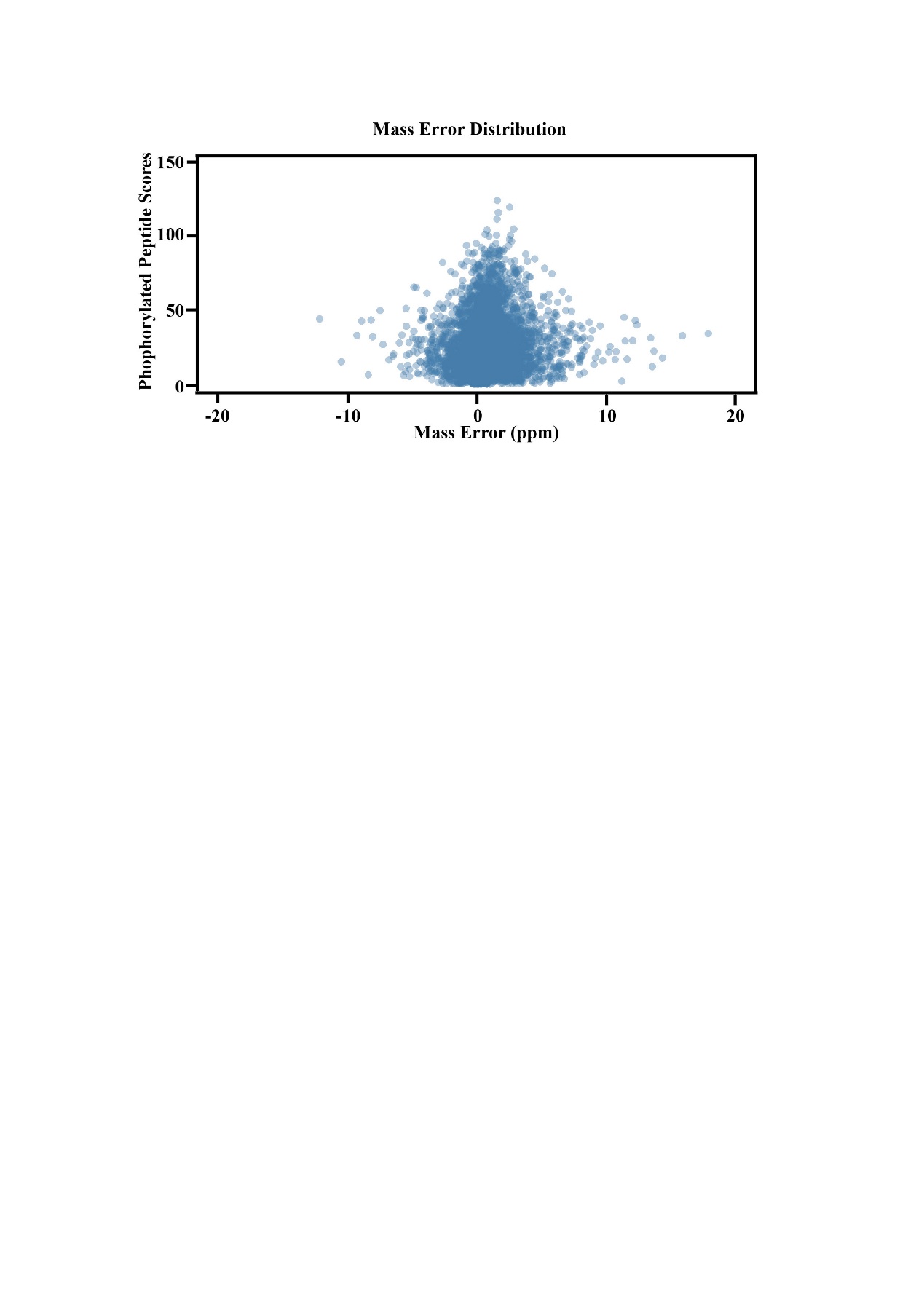


Figure S1 Mass error distribution of phosphorylated peptides. The x-axis represents the mass error in parts per million (ppm), and the y-axis shows the corresponding peptide scores. Each point indicates a phosphorylated peptide, with a dense cluster around zero mass error, reflecting high accuracy in peptide mass measurements.
