# Supplementary figures and images for: Hypovirus‐Induced Phosphorylation of CpIre1 Modulates Unfolded Protein Response and Virulence in Cryphonectria parasitica
Source: Mol Plant Pathol. 2026 Feb 15;27(2):e70227. doi: 10.1111/mpp.70227 (PMC12907514; doi:10.1111/mpp.70227)

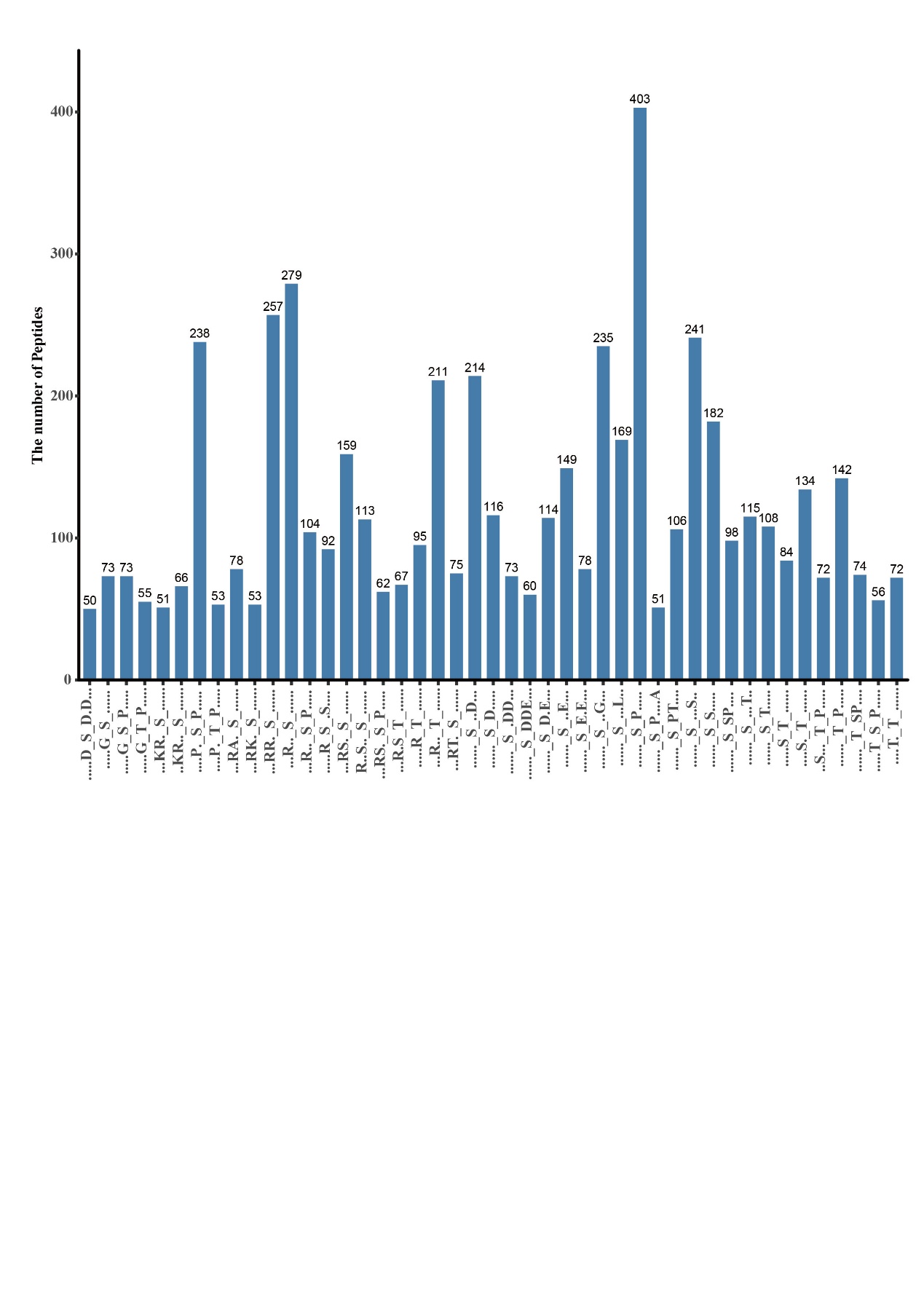


Figure S2. Number of identified peptides in each conserved motif.

Supplement: Supplementary file 2 — Figure S2: Number of identified peptides in each conserved motif. [file MPP-27-e70227-s010.docx]
