# Supplementary material for: Hypovirus‐Induced Phosphorylation of CpIre1 Modulates Unfolded Protein Response and Virulence in Cryphonectria parasitica
Source: Mol Plant Pathol. 2026 Feb 15;27(2):e70227. doi: 10.1111/mpp.70227 (PMC12907514; doi:10.1111/mpp.70227)
Supplement: Supplementary file 3 — Figure S3: The yeast two‐hybrid (Y2H) assay revealed no interaction between p29, p40, or p48 and CpIre1, respectively. [file MPP-27-e70227-s015.docx]

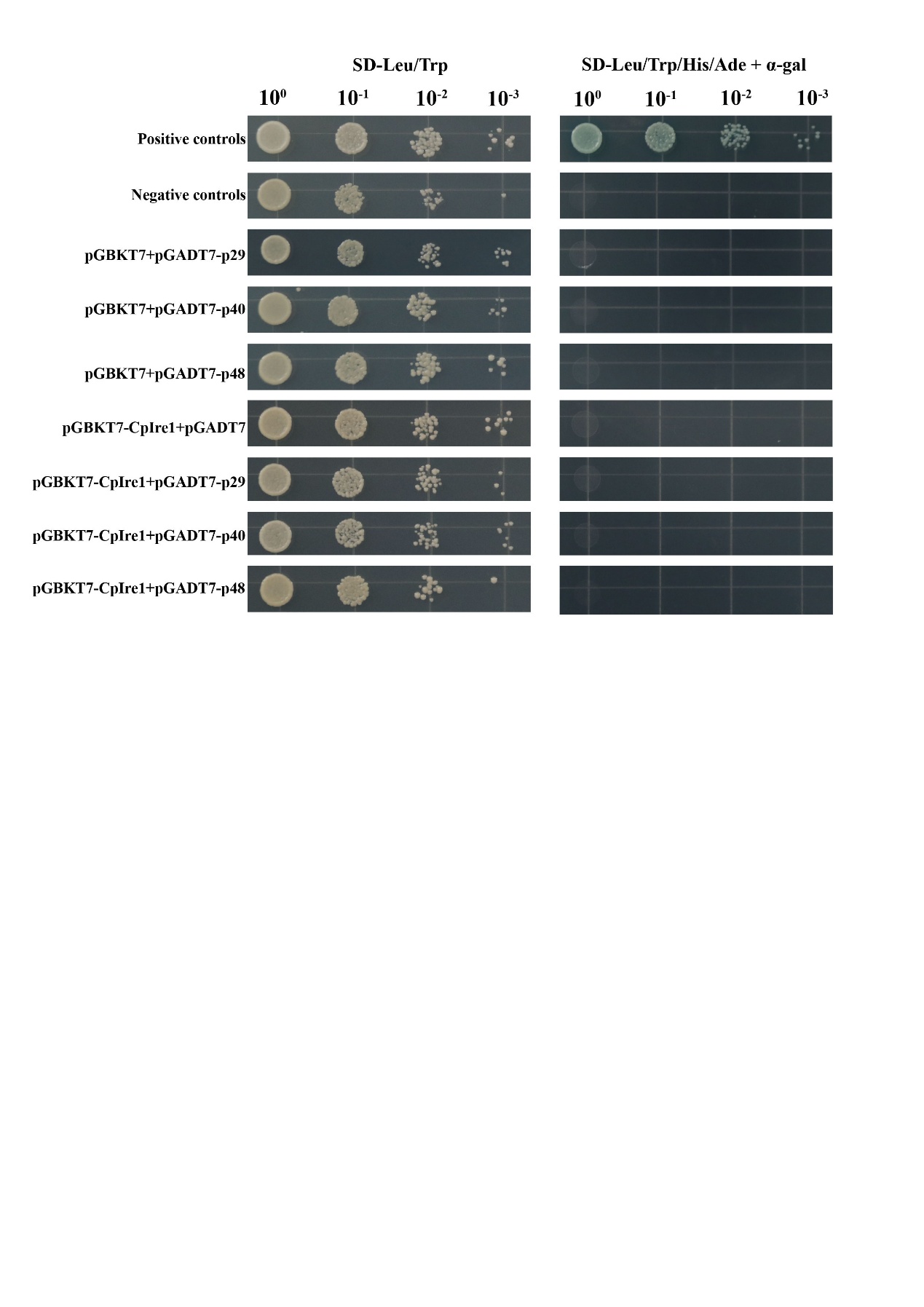


Figure S3. The yeast two-hybrid (Y2H) assay revealed no interaction between p29, p40, or p48 and CpIre1, respectively.
