# Supplementary material for: Hypovirus‐Induced Phosphorylation of CpIre1 Modulates Unfolded Protein Response and Virulence in Cryphonectria parasitica
Source: Mol Plant Pathol. 2026 Feb 15;27(2):e70227. doi: 10.1111/mpp.70227 (PMC12907514; doi:10.1111/mpp.70227)
Supplement: Supplementary file 4 — Figure S4: Heat and oxidative stress increase CpIre1 phosphorylation without altering total CpIre1 abundance. (a) Immunoblot analysis of phosphorylated CpIre1 (p‐CpIre1) and total CpIre1 in EP155 under control temperature (26°C), heat stress (30°C), or oxidative stress (1 mM H2O2). GAPDH was used as a loading control. (b) Quantification of CpIre1 phosphorylation level expressed as the p‐CpIre1/CpIre1 ratio. Data are presented as mean ± SD (n = 3). Different letters indicate statistically significant differences (p < 0.05). [file MPP-27-e70227-s013.docx]

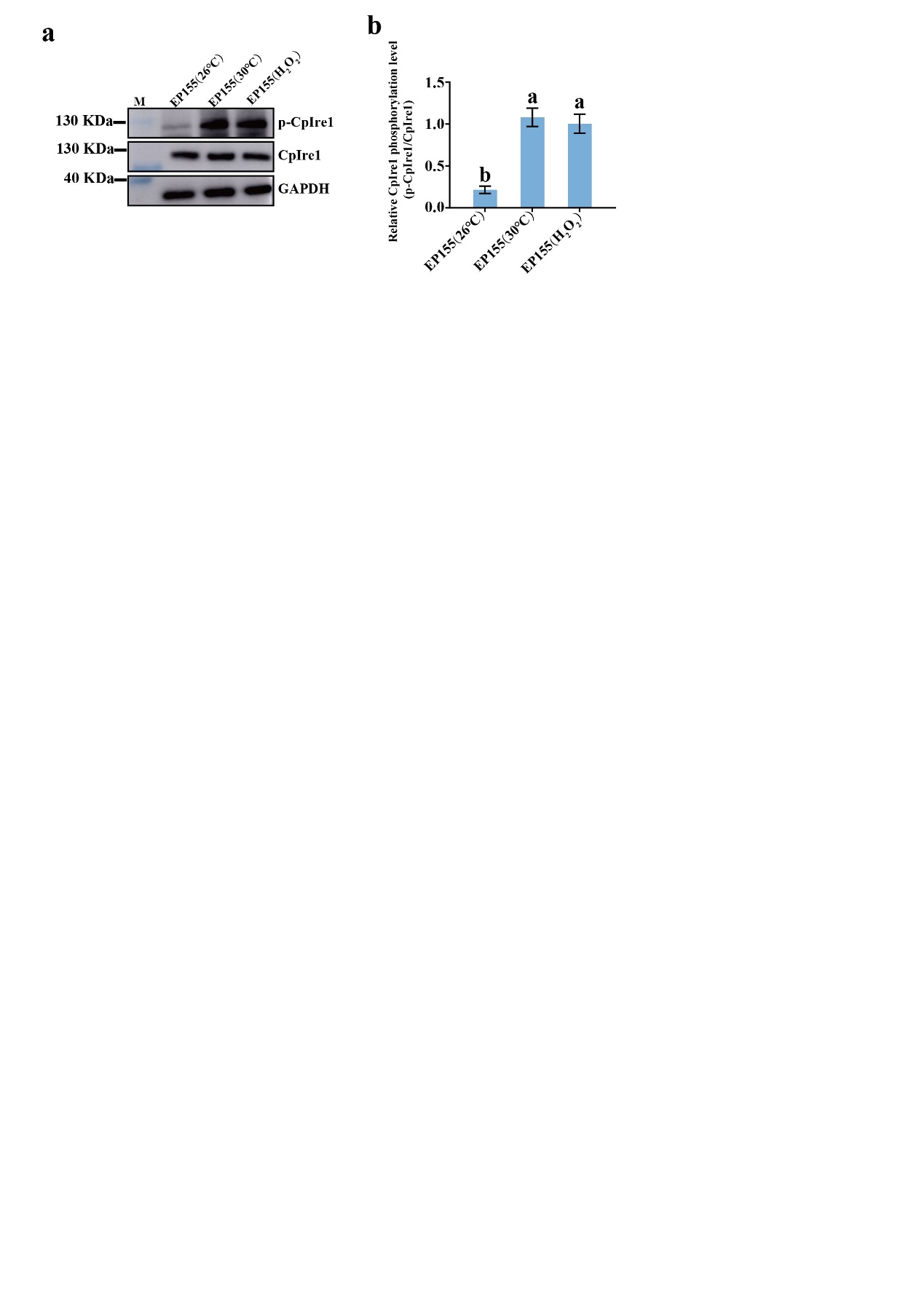


Figure S4. Heat and oxidative stress increase CpIre1 phosphorylation without altering total CpIre1 abundance. (a) Immunoblot analysis of phosphorylated CpIre1 (p-CpIre1) and total CpIre1 in EP155 under control temperature (26 °C), heat stress (30 °C), or oxidative stress (1mM H₂O₂). GAPDH was used as a loading control. (b) Quantification of CpIre1 phosphorylation level expressed as the p-CpIre1/CpIre1 ratio. Data are presented as mean ± SD (n=3). Different letters indicate statistically significant differences (*p* < 0.05).
