# Supplementary material for: Hypovirus‐Induced Phosphorylation of CpIre1 Modulates Unfolded Protein Response and Virulence in Cryphonectria parasitica
Source: Mol Plant Pathol. 2026 Feb 15;27(2):e70227. doi: 10.1111/mpp.70227 (PMC12907514; doi:10.1111/mpp.70227)
Supplement: Supplementary file 5 — Figure S5: Phylogenetic analysis and sequence similarity comparison of CpIre1 homologues across different species. (a) Phylogenetic tree of CpIre1 from various organisms generated with MEGAX software. Sequence similarity between CpIre1 and other homologous proteins was assessed using DNAMAN software to compare homologous protein sequences. (b) Sequence alignment of CpIre1 and its orthologs was conducted using CLC Genomics Workbench, with red boxes indicating conserved regions in CpIre1. The box indicates conserved amino acids. [file MPP-27-e70227-s002.docx]

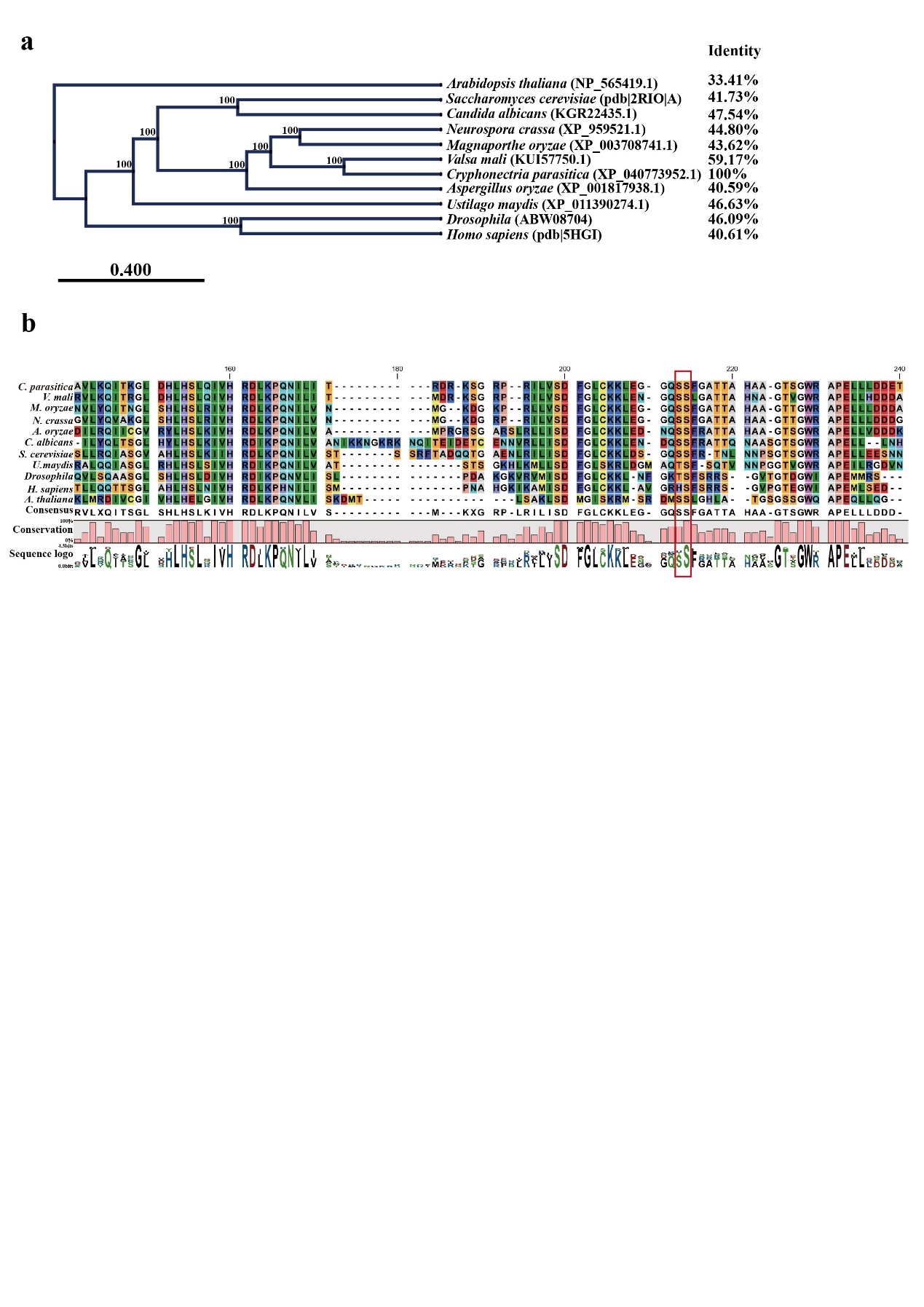


Figure S5. Phylogenetic analysis and sequence similarity comparison of CpIre1 homologs across different species. (a) Phylogenetic tree of CpIre1 from various organisms generated with MEGAX software. Sequence similarity between CpIre1 and other homologous proteins was assessed using DNAMAN software to compare homologous protein sequences. (b) Sequence alignment of CpIre1 and its orthologs was conducted using CLC Genomics Workbench, with red boxes indicating conserved regions in CpIre1. The box indicates conserved amino acids.
