# Supplementary material for: Hypovirus‐Induced Phosphorylation of CpIre1 Modulates Unfolded Protein Response and Virulence in Cryphonectria parasitica
Source: Mol Plant Pathol. 2026 Feb 15;27(2):e70227. doi: 10.1111/mpp.70227 (PMC12907514; doi:10.1111/mpp.70227)
Supplement: Supplementary file 6 — Figure S6: Generation of CpIre1 gene knockout strains. (a) Schematic representation of the CpIre1 homologous recombination construct. Lane 1: left arm of the CpIre1 gene; Lane 2: right arm; Lane 3: hph gene; Lane 4: fusion fragment containing the left arm, hph, and right arm. (b) PCR confirmation of CpIre1 gene knockout in mutant strains. (c) Schematic diagram of the CpIre1 gene deletion strategy. Probe A, targeting the hph gene, and Probe B, targeting the right arm, were used to distinguish wild‐type and ΔCpIre1 mutant strains based on fragment size in Southern blotting analysis. Scale bar = 1 kb. (d) Southern blotting analysis of ΔCpIre1 mutants, using Probe A (left) and Probe B (right). Genomic DNA from fungal strains was digested with Bsp1407 I, separated by agarose gel electrophoresis, and hybridised with Probe A and Probe B, respectively. [file MPP-27-e70227-s011.docx]

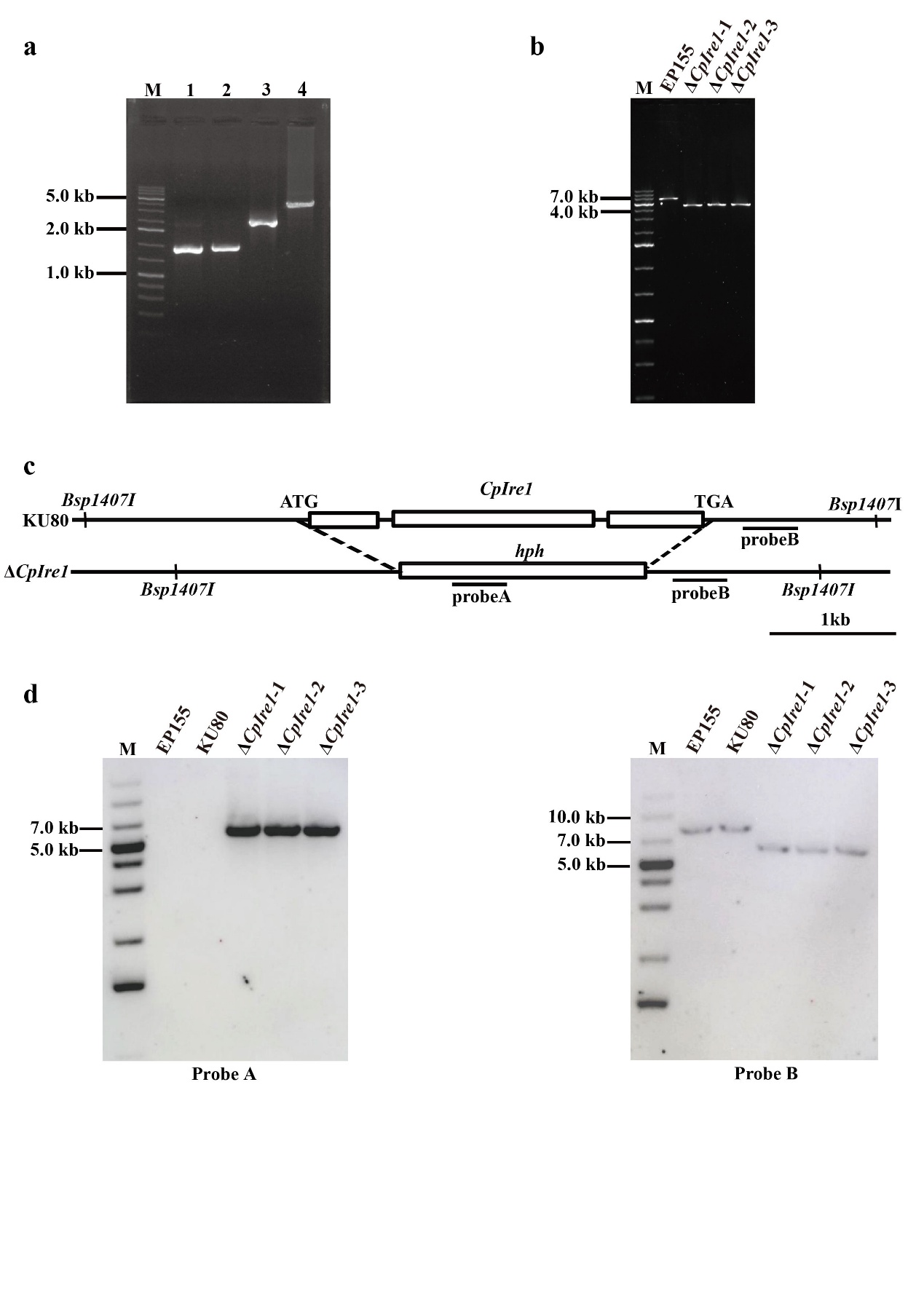


Figure S6. Generation of *CpIre1* gene knockout strains. (a) Schematic representation of the *CpIre1* homologous recombination construct. Lane 1: left arm of the *CpIre1* gene; Lane 2: right arm; Lane 3: *hph* gene; Lane 4: fusion fragment containing the left arm, *hph*, and right arm. (b) PCR confirmation of *CpIre1* gene knockout in mutant strains. (c) Schematic diagram of the *CpIre1* gene deletion strategy. Probe A, targeting the *hph* gene, and Probe B, targeting the right arm, were used to distinguish wild-type and Δ*CpIre1* mutant strains based on fragment size in Southern blotting analysis. Scale bar = 1 kb. (d) Southern blotting analysis of Δ*CpIre1* mutants, using Probe A (left) and Probe B (right). Genomic DNA from fungal strains was digested with *Bsp1407* I, separated by agarose gel electrophoresis, and hybridized with Probe A and Probe B, respectively.
