# Supplementary material for: Hypovirus‐Induced Phosphorylation of CpIre1 Modulates Unfolded Protein Response and Virulence in Cryphonectria parasitica
Source: Mol Plant Pathol. 2026 Feb 15;27(2):e70227. doi: 10.1111/mpp.70227 (PMC12907514; doi:10.1111/mpp.70227)
Supplement: Supplementary file 7 — Figure S7: Construction and validation of the complementation strain and point mutation strains of CpIre1. (a) Diagram outlining the construction of the CpIre1 gene complementation plasmid, pCPXG418‐com‐CpIre1. (b) Validation of the pCPXG418‐com‐CpIre1 plasmid by EcoR I restriction enzyme digestion. Lane 1: CpIre1 gene. Lane 2: pCPXG418 plasmid digested with EcoR I. Lane 3: pCPXG418‐com‐CpIre1 plasmid. Lane 4: pCPXG418‐com‐CpIre1 plasmid digested with EcoR I. (c) PCR confirmation of ΔCpIre1‐com. (d) Southern blotting analysis of ΔCpIre1‐com, using Probe B (right) (Figure S6c). (e) DNA sequencing results confirmed the construction of the point mutation plasmids. [file MPP-27-e70227-s014.docx]

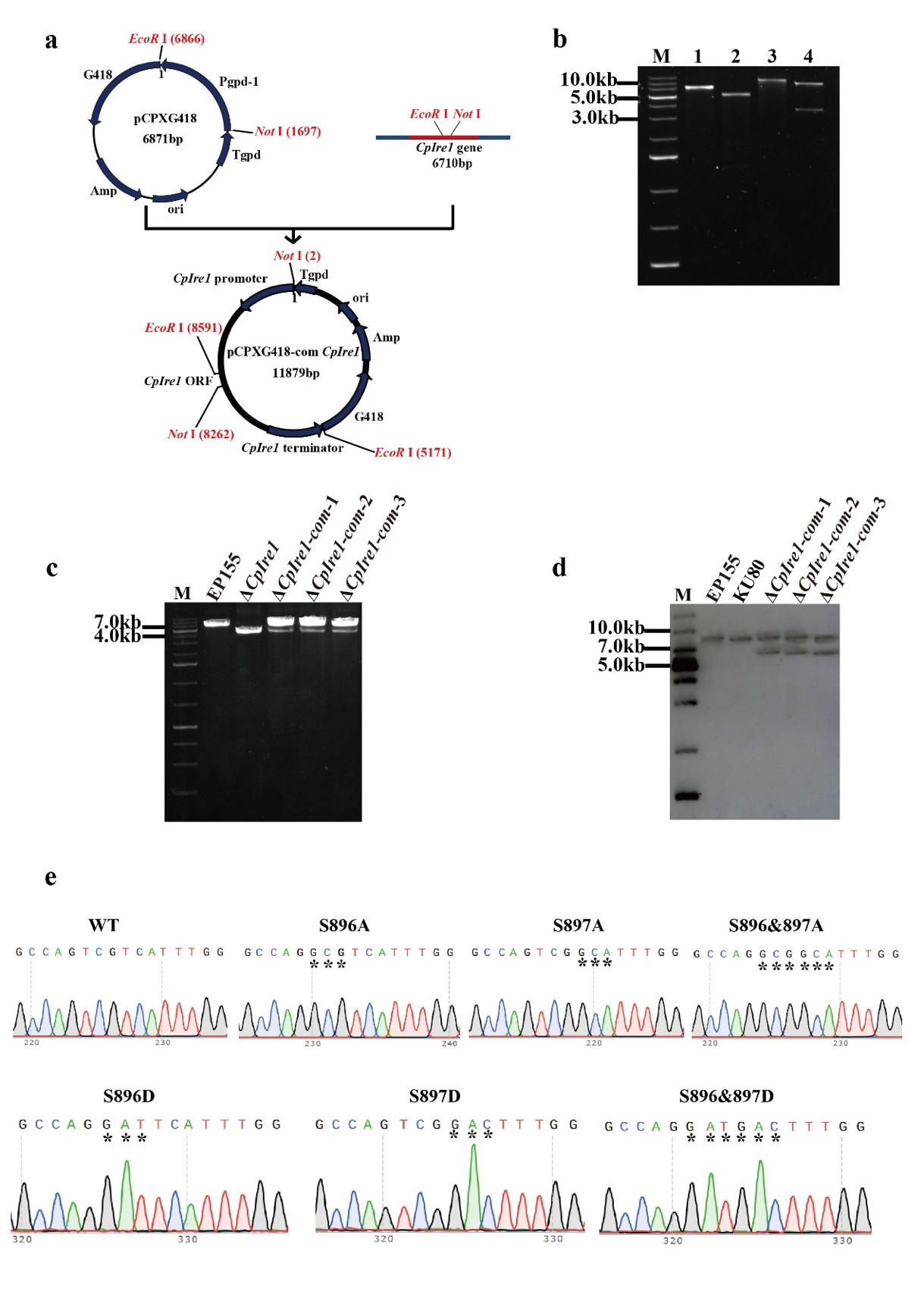


Figure S7. Construction and validation of the complementation strain and point mutation strains of *CpIre1*. (a) Diagram outlining the construction of the *CpIre1* gene complementation plasmid, pCPXG418-com-*CpIre1*. (b) Validation of the pCPXG418-com-*CpIre1* plasmid by *EcoR* I restriction enzyme digestion. Lane 1: *CpIre1* gene. Lane 2: pCPXG418 plasmid digested with *EcoR* I. Lane 3: pCPXG418-com-*CpIre1* plasmid. Lane 4: pCPXG418-com-*CpIre1* plasmid digested with *EcoR* I. (c) PCR confirmation of Δ*CpIre1*-com. (d) Southern blotting analysis of Δ*CpIre1*-com, using Probe B (right) (Figure S6c). (e) DNA sequencing results confirmed the construction of the point mutation plasmids.
