# Supplementary material for: Hypovirus‐Induced Phosphorylation of CpIre1 Modulates Unfolded Protein Response and Virulence in Cryphonectria parasitica
Source: Mol Plant Pathol. 2026 Feb 15;27(2):e70227. doi: 10.1111/mpp.70227 (PMC12907514; doi:10.1111/mpp.70227)
Supplement: Supplementary file 8 — Figure S8: Growth of fungal strains on PDA medium supplemented with H2O2, SDS, NaCl, or Congo red. (a) Colony morphology of wild‐type, deletion mutant, complementation mutant, and overexpression mutant strains after 7 days of incubation at 26°C. Scale bar = 2 cm. (b) Growth inhibition rate of the strains in response to stress conditions, with colony diameter on standard PDA set as 100%. All measurements were taken after 7 days at 26°C and performed in triplicate. Error bars represent the standard deviation from three independent biological replicates. There are significant differences between samples indicated by different letters on the bars (ANOVA followed by Tukey's test, p < 0.05). [file MPP-27-e70227-s016.docx]

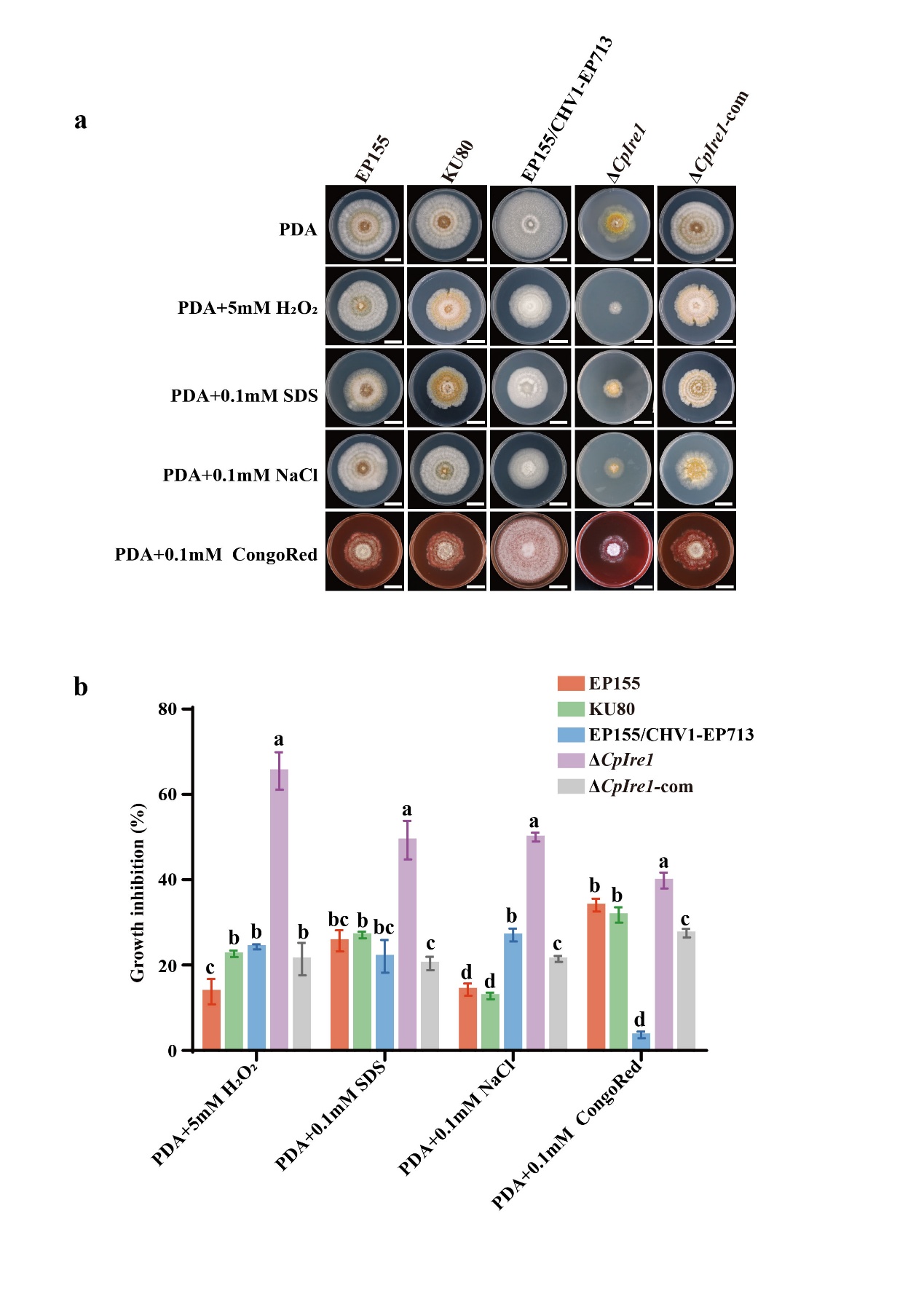


Figure S8. Growth of fungal strains on PDA medium supplemented with H_2_O_2_, SDS, NaCl, or Congo red. (a) Colony morphology of wild-type, deletion mutant, complementation mutant, and overexpression mutant strains after 7 d of incubation at 26°C. Scale bar = 2 cm. (b) Growth inhibition rate of the strains in response to stress conditions, with colony diameter on standard PDA set as 100%. All measurements were taken after 7 days at 26°C and performed in triplicate. Error bars represent the standard deviation from three independent biological replicates. There are significant differences between samples indicated by different letters on the bars (ANOVA followed by Tukey’s test, *p*<0.05).
