# Supplementary material for: Hypovirus‐Induced Phosphorylation of CpIre1 Modulates Unfolded Protein Response and Virulence in Cryphonectria parasitica
Source: Mol Plant Pathol. 2026 Feb 15;27(2):e70227. doi: 10.1111/mpp.70227 (PMC12907514; doi:10.1111/mpp.70227)
Supplement: Supplementary file 9 — Figure S9: Characterisation of colony morphology, sporulation, and virulence in phospho‐mimic CpIre1 mutant strains. (a) Colony morphology of the mutants on PDA medium. Photographs were taken at 7 and 14 days after inoculation. Scale bar = 2 cm. (b) Mutant colony areas were measured at Day 7 and 14 post‐inoculation, respectively. (c) Sporulation levels of the tested strains. Spores were harvested and counted on Day 14 post‐inoculation. (d) Red Fuji apples were inoculated with the tested strains, maintained at 26°C, and were measured and photographed on Day 10 post‐inoculation. Letters above the columns indicate statistical significance of the difference between three treatments (ANOVA followed by Tukey's test, p < 0.05). [file MPP-27-e70227-s017.docx]

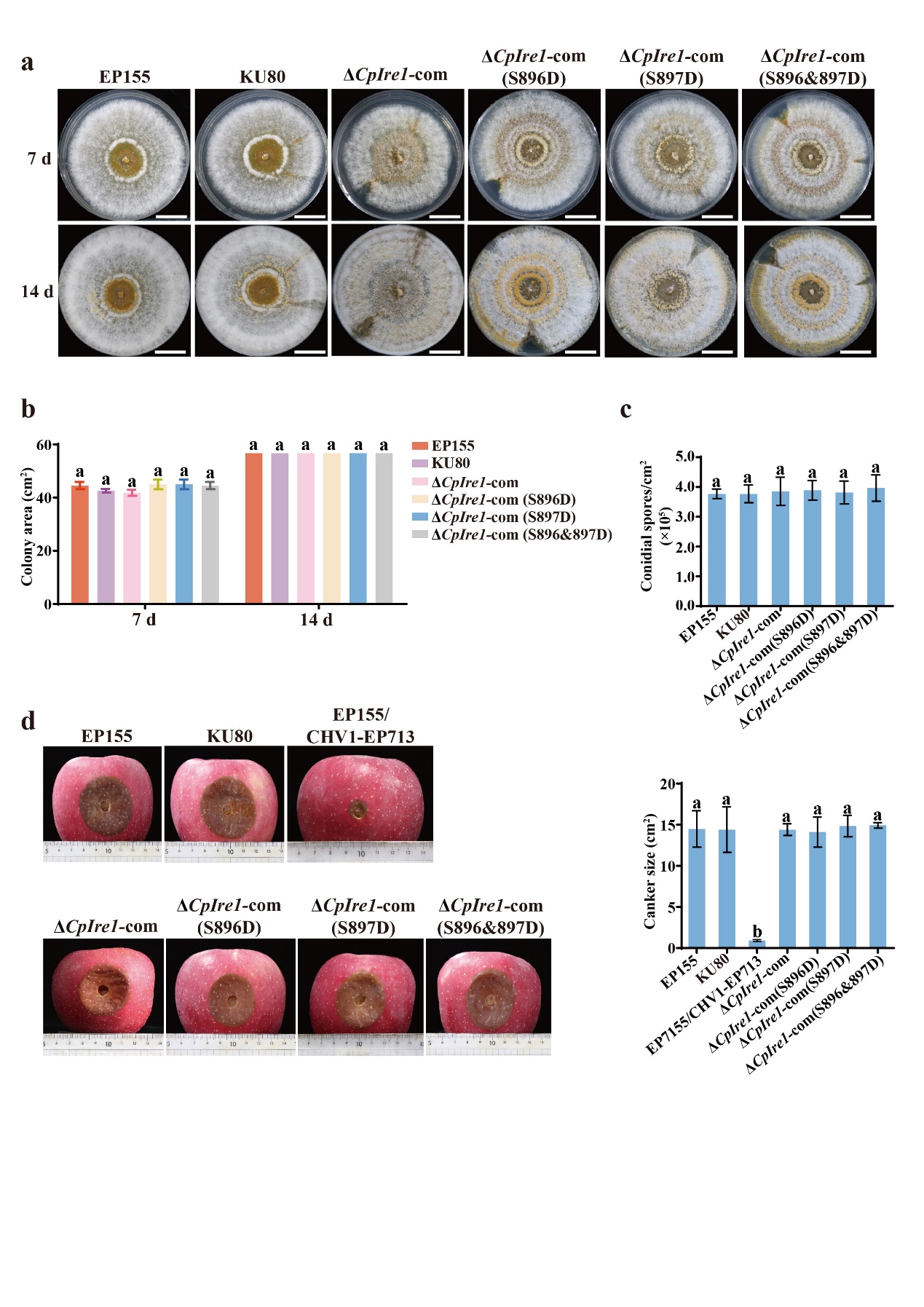


Figure S9. Characterization of colony morphology, sporulation, and virulence in phospho-mimic *CpIre1* mutant strains. (a) Colony morphology of the mutants on PDA medium. Photographs were taken at 7 and 14 days after inoculation. Scale bar = 2 cm. (b) Mutant colony areas were measured at day 7 and 14 post-inoculation, respectively. (c) Sporulation levels of the tested strains. Spores were harvested and counted on day 14 post-inoculation. (d) Red Fuji apples were inoculated with the tested strains, maintained at 26°C, and were measured and photographed on day 10 post-inoculation. Letters above the columns indicate statistical significance of the difference between three treatments (ANOVA followed by Tukey’s test, *p* < 0.05).
