# Supplementary material for: Hypovirus‐Induced Phosphorylation of CpIre1 Modulates Unfolded Protein Response and Virulence in Cryphonectria parasitica
Source: Mol Plant Pathol. 2026 Feb 15;27(2):e70227. doi: 10.1111/mpp.70227 (PMC12907514; doi:10.1111/mpp.70227)
Supplement: Supplementary file 10 — Figure S10: Colony morphology and viral dsRNA accumulation were examined in hypovirus‐containing CpIre1 phospho‐mimic mutants. (a) Colony morphology of hypovirus‐free and hypovirus‐containing CpIre1 phospho‐mimic mutants grown on PDA for 7 days. Scale bar = 2 cm. (b) Viral dsRNA accumulation in hypovirus‐containing CpIre1 phospho‐mimic mutants, as analysed by agarose gel electrophoresis. (c) Viral RNA levels in the infected strains were determined by qRT‐PCR. Error bars represent standard deviations from three independent biological replicates. Significant differences between samples (indicated by different letters) were determined by (ANOVA followed by Tukey's test p < 0.05). [file MPP-27-e70227-s008.docx]

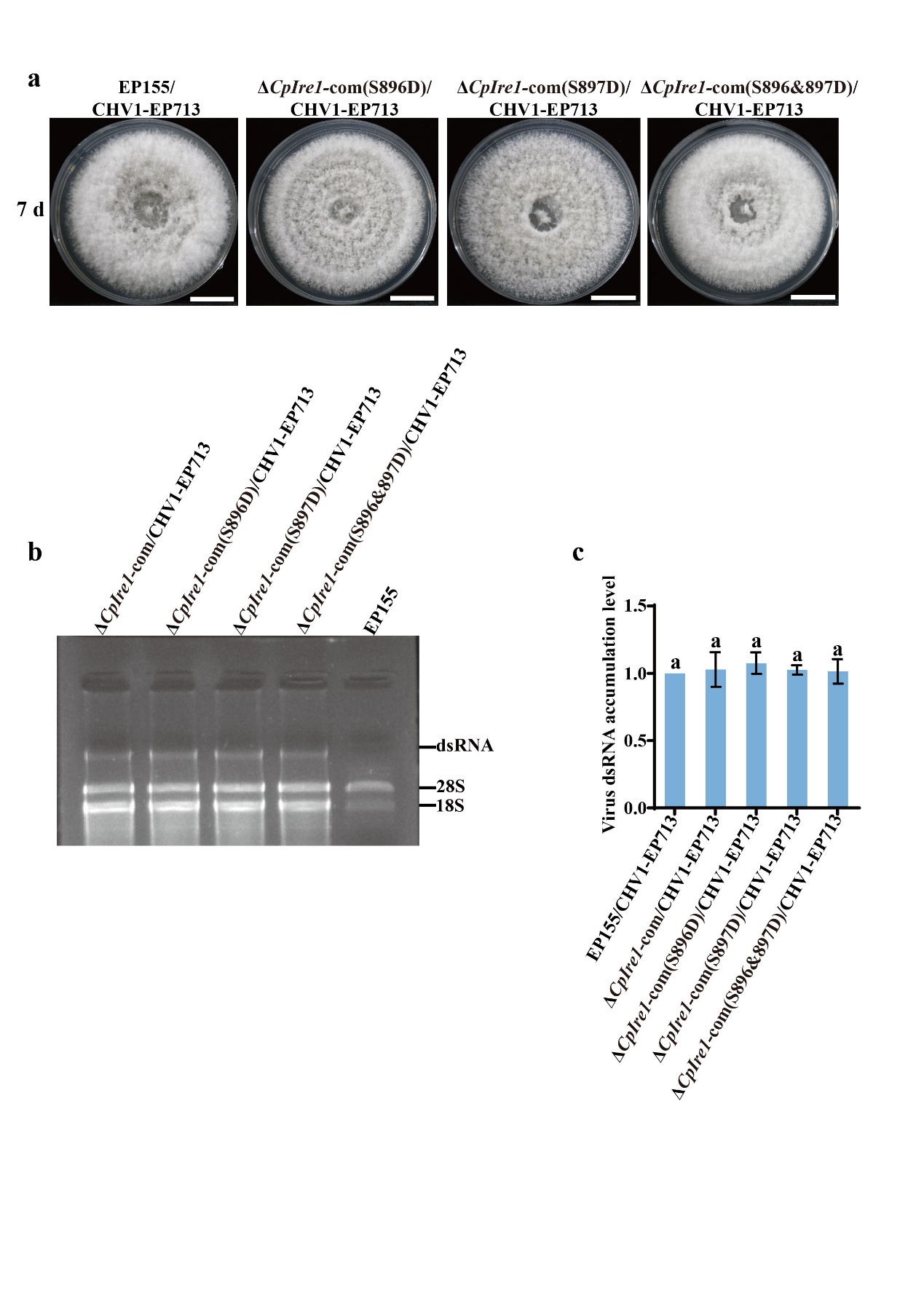


Figure S10. Colony morphology and viral dsRNA accumulation were examined in hypovirus-containing *CpIre1* phospho-mimic mutants. (a) Colony morphology of hypovirus-free and hypovirus-containing CpIre1 phospho-mimic mutants grown on PDA for 7 days. Scale bar = 2 cm. (b) Viral dsRNA accumulation in hypovirus-containing *CpIre1* phospho-mimic mutants, as analyzed by agarose gel electrophoresis. (c) Viral RNA levels in the infected strains were determined by qRT-PCR. Error bars represent standard deviations from three independent biological replicates. Significant differences between samples (indicated by different letters) were determined by (ANOVA followed by Tukey's test *p* < 0.05).
