# Supplementary material for: Hypovirus‐Induced Phosphorylation of CpIre1 Modulates Unfolded Protein Response and Virulence in Cryphonectria parasitica
Source: Mol Plant Pathol. 2026 Feb 15;27(2):e70227. doi: 10.1111/mpp.70227 (PMC12907514; doi:10.1111/mpp.70227)
Supplement: Supplementary file 11 — Figure S11: Virulence analysis of the CpIre1 complementation strain, phospho‐deficient mutants, and phospho‐mimic mutants infected with CHV1‐EP713. (a) Red Fuji apples were inoculated with the tested strains, maintained at 26°C, and photographed on Day 10 post‐inoculation. (b) Measurement of canker development in different fungal strains. Letters above the columns indicate statistical significance of the difference between three treatments (ANOVA followed by Tukey's test, p < 0.05). [file MPP-27-e70227-s005.docx]

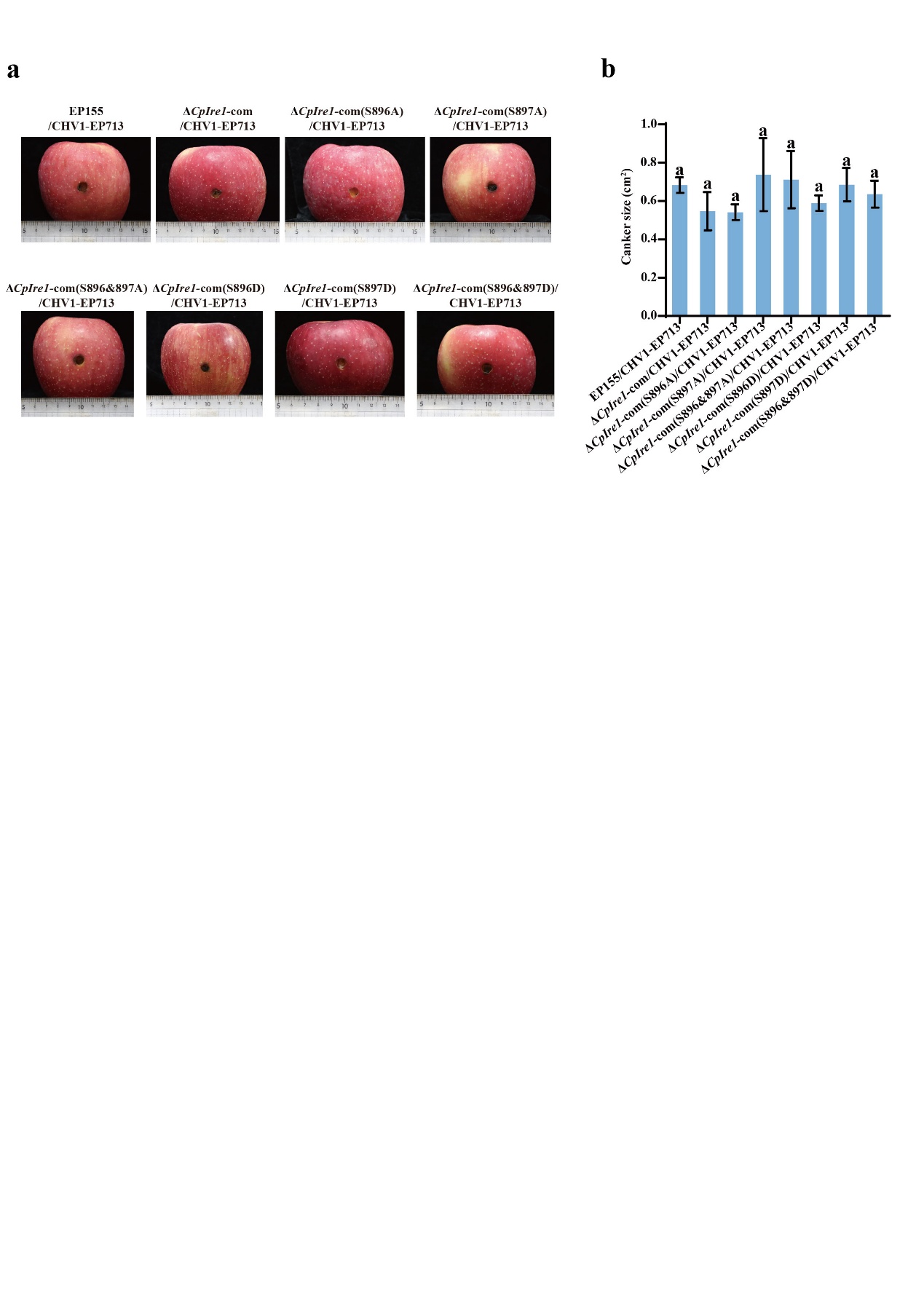


Figure S11. Virulence analysis of the *CpIre1* complementation strain, phospho-deficient mutants, and phospho-mimic mutants infected with CHV1-EP713. (a) Red Fuji apples were inoculated with the tested strains, maintained at 26°C, and photographed on day 10 post-inoculation. (b) Measurement of canker development in different fungal strains. Letters above the columns indicate statistical significance of the difference between three treatments (ANOVA followed by Tukey’s test, *p* < 0.05).
