# Supplementary material for: Hypovirus‐Induced Phosphorylation of CpIre1 Modulates Unfolded Protein Response and Virulence in Cryphonectria parasitica
Source: Mol Plant Pathol. 2026 Feb 15;27(2):e70227. doi: 10.1111/mpp.70227 (PMC12907514; doi:10.1111/mpp.70227)
Supplement: Supplementary file 12 — Figure S12: RNA‐seq analysis reveals the impact of CpIre1 deletion on gene expression in CHV1‐EP713‐infected C. parasitica . (a) Heatmap displaying the relative gene expression levels in ΔCpIre1/CHV1‐EP713 compared to the KU80/CHV1‐EP713 strain, based on RNA‐seq data. (b) Volcano plot illustrating DEGs between KU80/CHV1‐EP713 and ΔCpIre1/CHV1‐EP713. mRNAs with log2FC > 1 in ΔCpIre1 relative to KU80 (p < 0.01, two‐tailed t‐test) are marked in red, while mRNAs with log2FC < −1 (p < 0.01, two‐tailed t‐test) are highlighted in blue. (c) GO enrichment analysis of DEGs, categorised by biological process (BP), cellular component (CC), and molecular function (MF). (d) KEGG pathway enrichment analysis of the DEGs. DEG refers to differentially expressed gene. [file MPP-27-e70227-s012.docx]

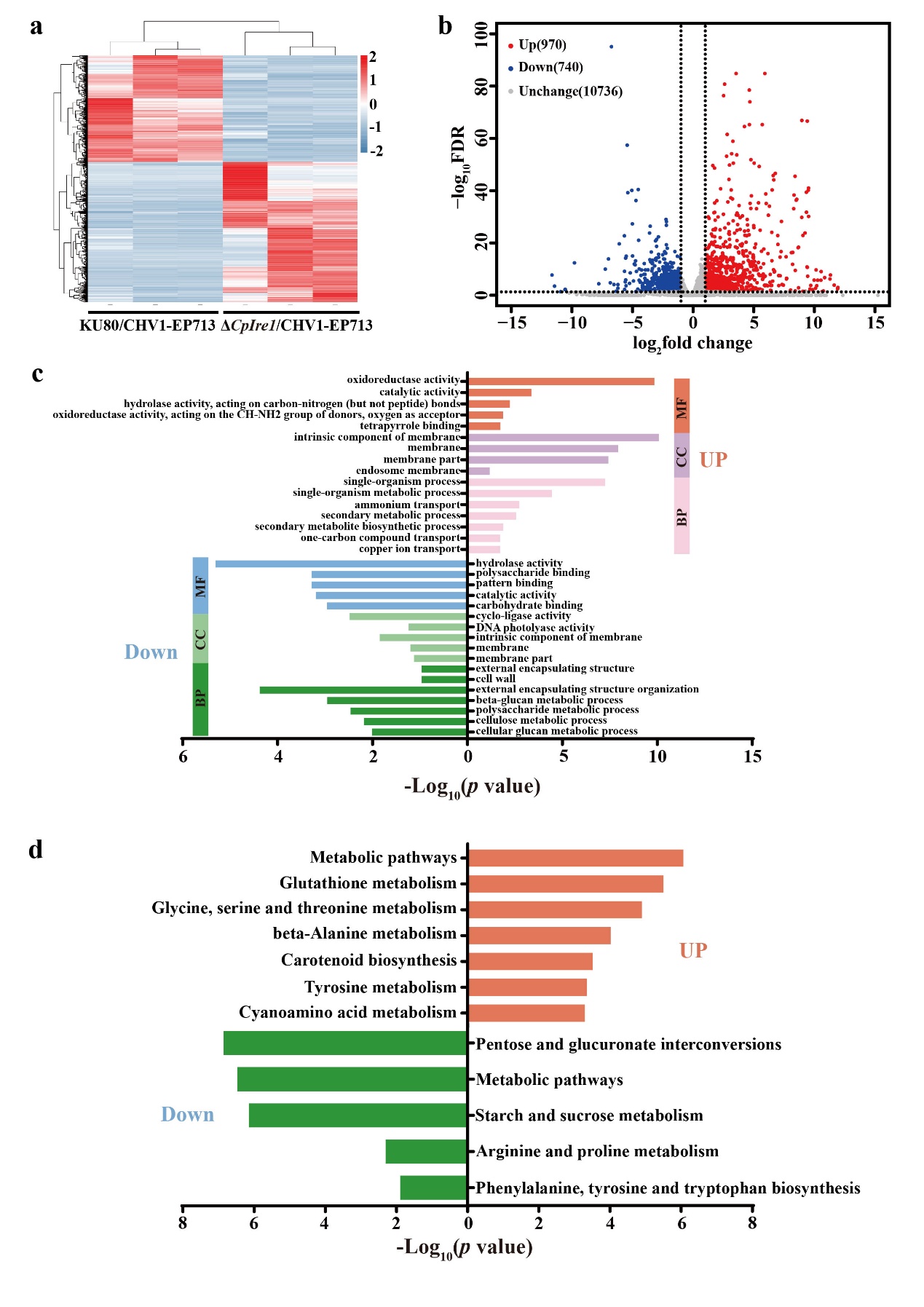


Figure S12. RNA-seq analysis reveals the impact of *CpIre1* deletion on gene expression in CHV1-EP713-infected *C. parasitica*. (a) Heatmap displaying the relative gene expression levels in Δ*CpIre1*/CHV1-EP713 compared to the KU80/CHV1-EP713 strain, based on RNA-seq data. (b) Volcano plot illustrating DEGs between KU80/CHV1-EP713 and Δ*CpIre1*/CHV1-EP713. mRNAs with log_2_FC >1 in Δ*CpIre1* relative to KU80 (*p* < 0.01, two-tailed t-test) are marked in red, while mRNAs with log_2_FC < -1 (*p* < 0.01, two-tailed t-test) are highlighted in blue. (c) GO enrichment analysis of DEGs, categorized by biological process (BP), cellular component (CC), and molecular function (MF). (d) KEGG pathway enrichment analysis of the DEGs. DEG refers to differentially expressed gene.
