# Supplementary material for: Hypovirus‐Induced Phosphorylation of CpIre1 Modulates Unfolded Protein Response and Virulence in Cryphonectria parasitica
Source: Mol Plant Pathol. 2026 Feb 15;27(2):e70227. doi: 10.1111/mpp.70227 (PMC12907514; doi:10.1111/mpp.70227)
Supplement: Supplementary file 14 — Table S2: Analysis of the phosphorylation‐modified peptide motif. [file MPP-27-e70227-s006.docx]

**Table S2 Analysis of the phosphorylation-modified peptide motif (occurrences = 50, significance = 0.00018, background = Cparasiticav2.GeneCatalog20091217.proteins-2)**

| **#** | **Motif Logo** | **Motif** | **Score** | **Foreground Matches** | **Foreground Size** | **Background Matches** | **Background Size** | **Fold Increase** |
| --- | --- | --- | --- | --- | --- | --- | --- | --- |
| 1 | 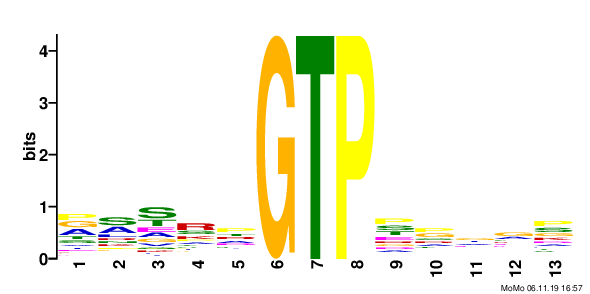 | .....G_T_P..... | 28.76 | 55 | 1358 | 8 | 1358 | 6.9 |
| 2 | 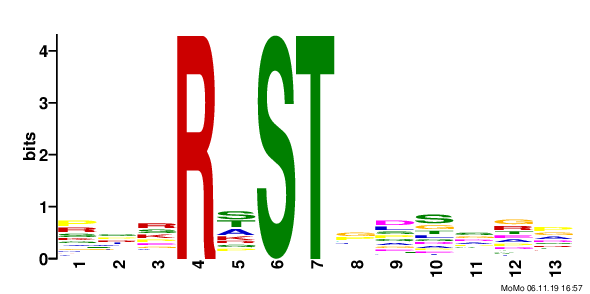 | ...R.S_T_...... | 25.48 | 67 | 1303 | 13 | 1350 | 5.3 |
| 3 | 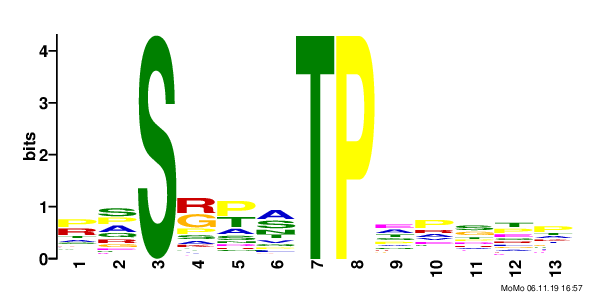 | ..S..._T_P..... | 22.08 | 72 | 1236 | 21 | 1337 | 3.7 |
| 4 | 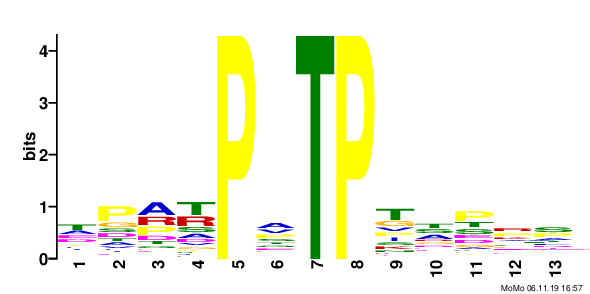 | ....P._T_P..... | 20.49 | 53 | 1164 | 18 | 1316 | 3.3 |
| 5 | 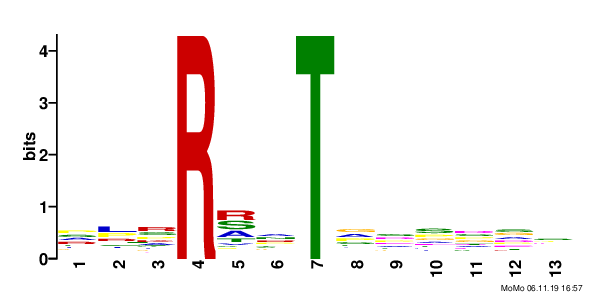 | ...R.._T_...... | 16.00 | 211 | 1111 | 117 | 1298 | 2.1 |
| 6 | 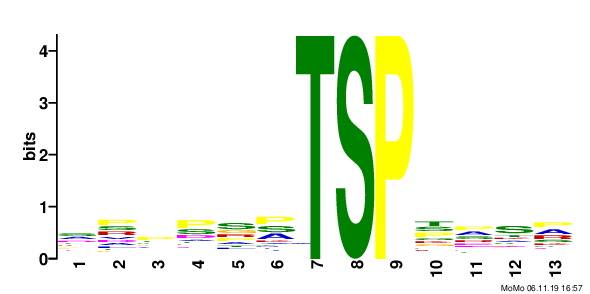 | ......_T_SP.... | 26.71 | 74 | 900 | 25 | 1181 | 3.9 |
| 7 | 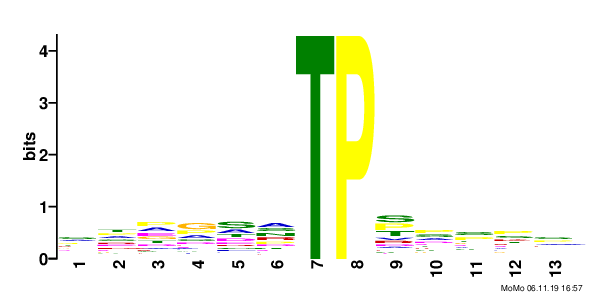 | ......_T_P..... | 14.16 | 142 | 826 | 100 | 1156 | 2.0 |
| 8 | 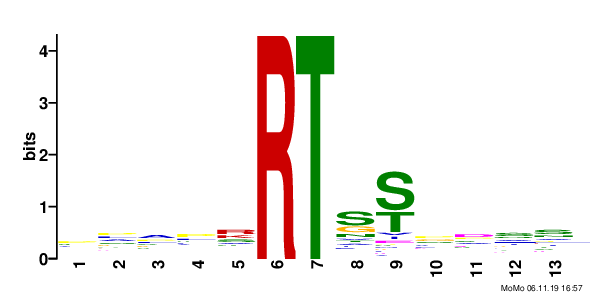 | .....R_T_...... | 10.86 | 95 | 684 | 70 | 1056 | 2.1 |
| 9 | 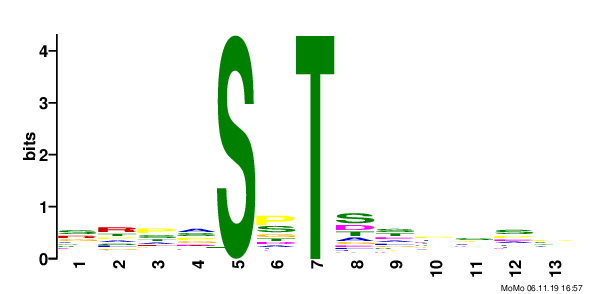 | ....S._T_...... | 7.59 | 134 | 589 | 140 | 986 | 1.6 |
| 10 | 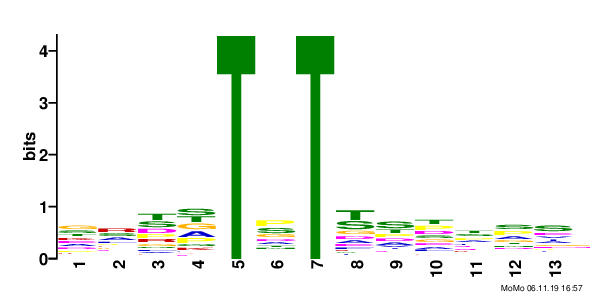 | ....T._T_...... | 5.38 | 72 | 455 | 77 | 846 | 1.7 |
| 11 | 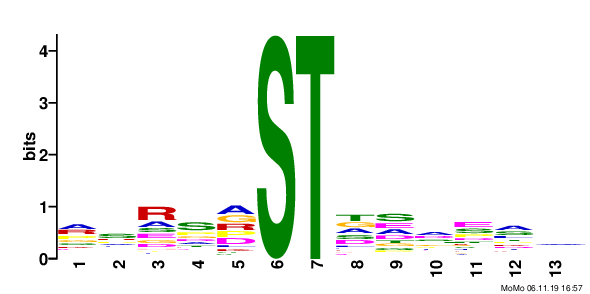 | .....S_T_...... | 4.36 | 84 | 383 | 110 | 769 | 1.5 |
| 12 | 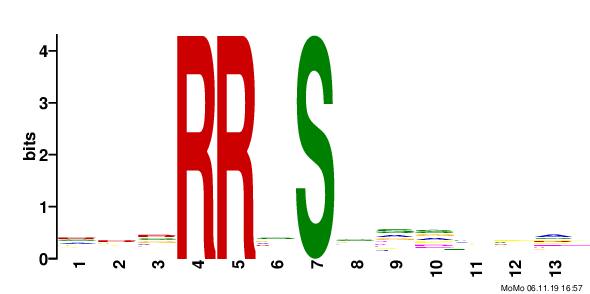 | ...RR._S_...... | 32.00 | 257 | 4866 | 54 | 4866 | 4.8 |
| 13 | 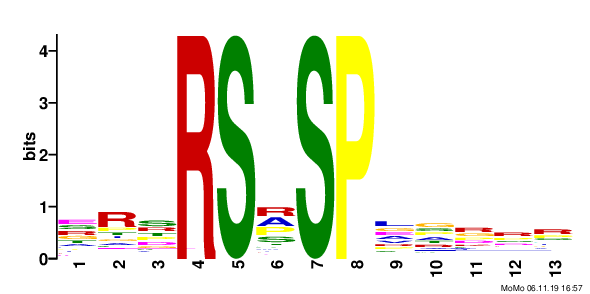 | ...RS._S_P..... | 37.09 | 62 | 4609 | 8 | 4812 | 8.1 |
| 14 | 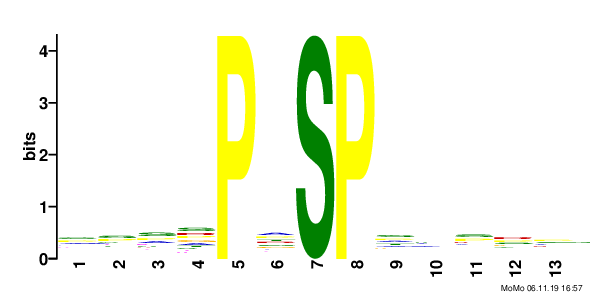 | ....P._S_P..... | 28.91 | 238 | 4547 | 66 | 4804 | 3.8 |
| 15 | 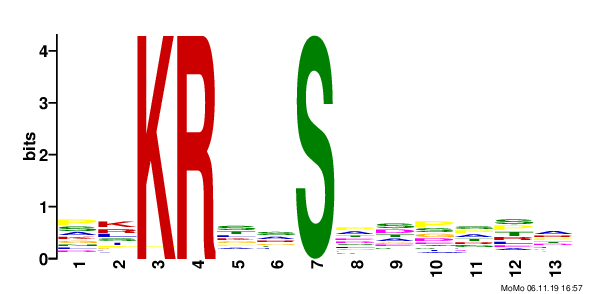 | ..KR.._S_...... | 28.36 | 66 | 4309 | 12 | 4738 | 6.0 |
| 16 | 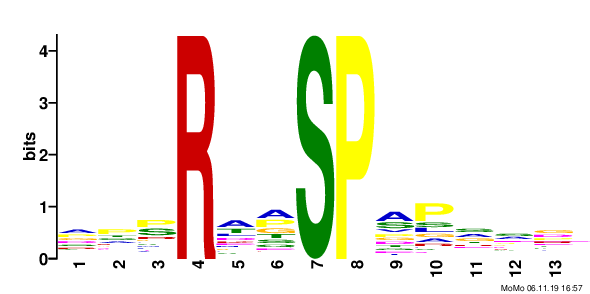 | ...R.._S_P..... | 26.37 | 104 | 4243 | 26 | 4726 | 4.5 |
| 17 | 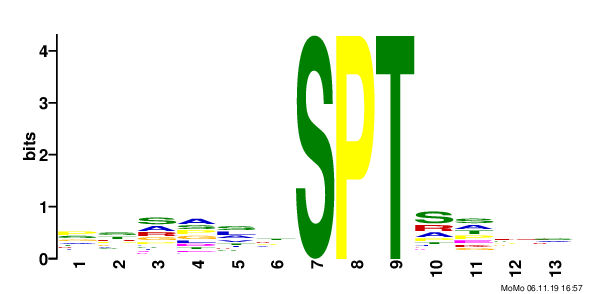 | ......_S_PT.... | 25.43 | 106 | 4139 | 30 | 4700 | 4.0 |
| 18 | 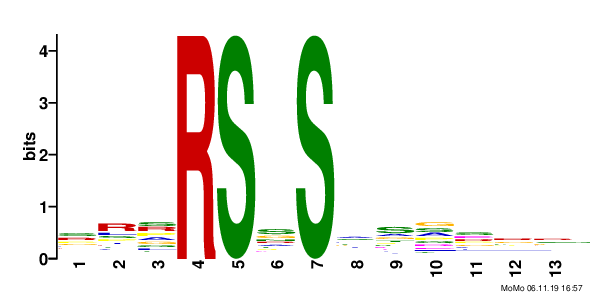 | ...RS._S_...... | 25.12 | 159 | 4033 | 57 | 4670 | 3.2 |
| 19 | 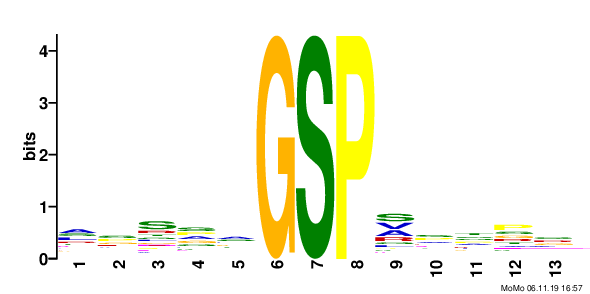 | .....G_S_P..... | 23.79 | 73 | 3874 | 21 | 4613 | 4.1 |
| 20 | 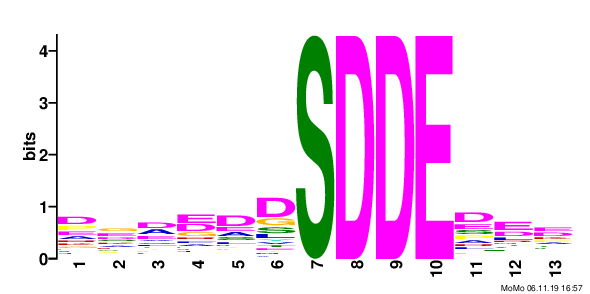 | ......_S_DDE... | 47.36 | 60 | 3801 | 5 | 4592 | 14.5 |
| 21 | 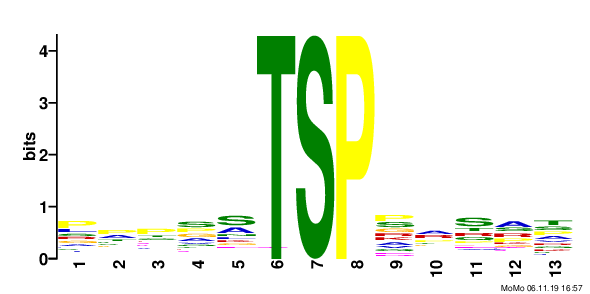 | .....T_S_P..... | 23.15 | 56 | 3741 | 16 | 4587 | 4.3 |
| 22 | 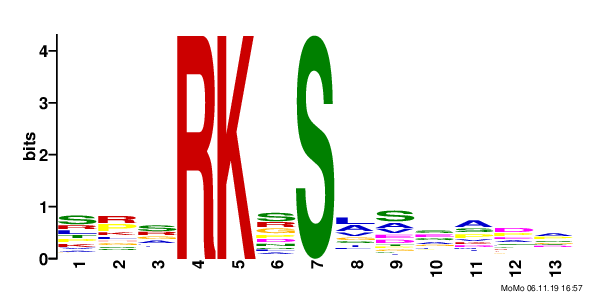 | ...RK._S_...... | 26.84 | 53 | 3685 | 12 | 4571 | 5.5 |
| 23 | 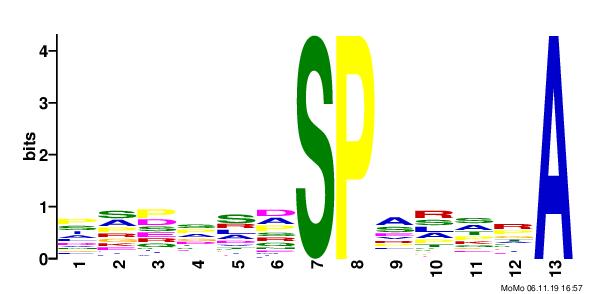 | ......_S_P....A | 20.17 | 51 | 3632 | 19 | 4559 | 3.4 |
| 24 | 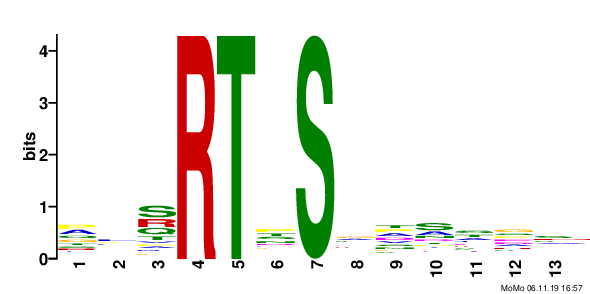 | ...RT._S_...... | 24.99 | 75 | 3581 | 25 | 4540 | 3.8 |
| 25 | 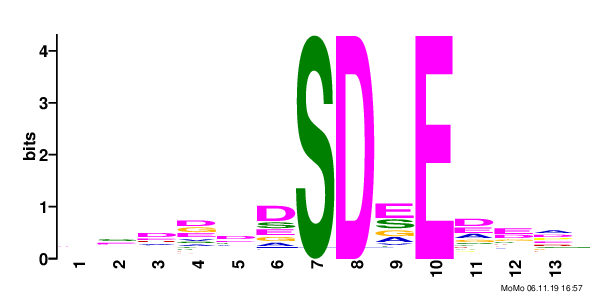 | ......_S_D.E... | 30.64 | 114 | 3506 | 40 | 4515 | 3.7 |
| 26 | 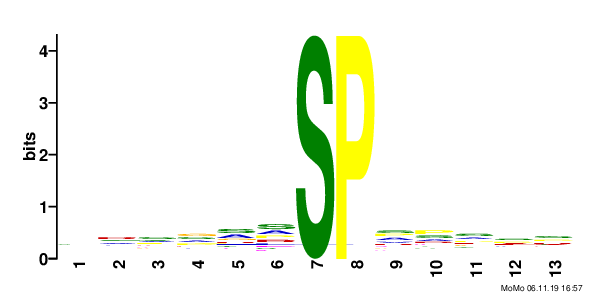 | ......_S_P..... | 16.00 | 403 | 3392 | 281 | 4475 | 1.9 |
| 27 | 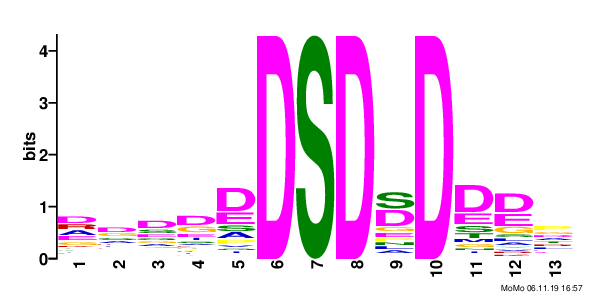 | .....D_S_D.D... | 28.01 | 50 | 2989 | 16 | 4194 | 4.4 |
| 28 | 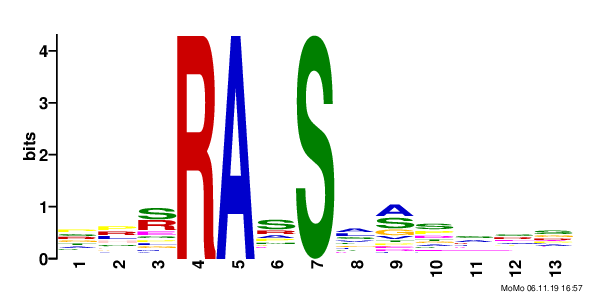 | ...RA._S_...... | 25.37 | 78 | 2939 | 29 | 4178 | 3.8 |
| 29 | 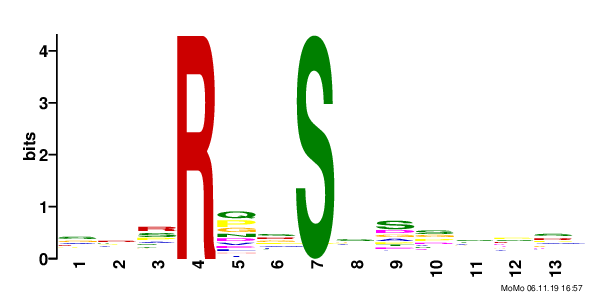 | ...R.._S_...... | 14.65 | 279 | 2861 | 247 | 4149 | 1.6 |
| 30 | 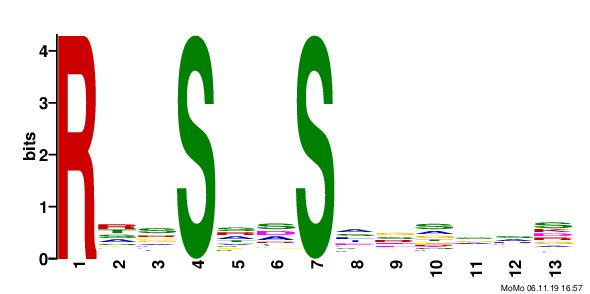 | R..S.._S_...... | 29.55 | 113 | 2582 | 55 | 3902 | 3.1 |
| 31 | 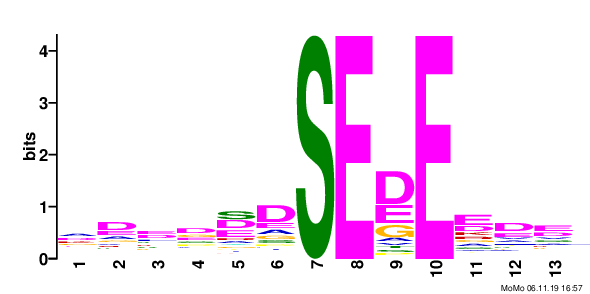 | ......_S_E.E... | 24.17 | 78 | 2469 | 34 | 3847 | 3.6 |
| 32 | 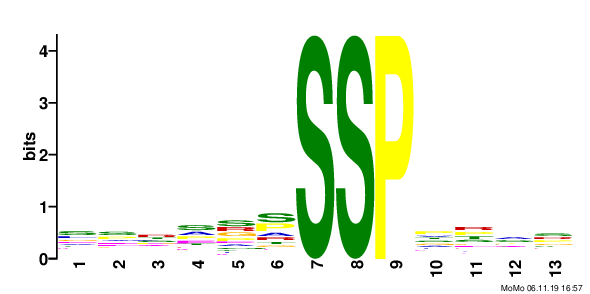 | ......_S_SP.... | 24.57 | 98 | 2391 | 55 | 3813 | 2.8 |
| 33 | 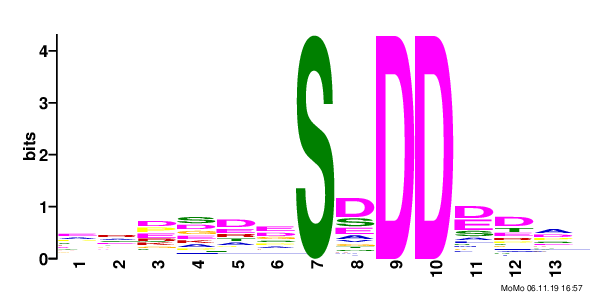 | ......_S_.DD... | 18.38 | 73 | 2293 | 47 | 3758 | 2.5 |
| 34 | 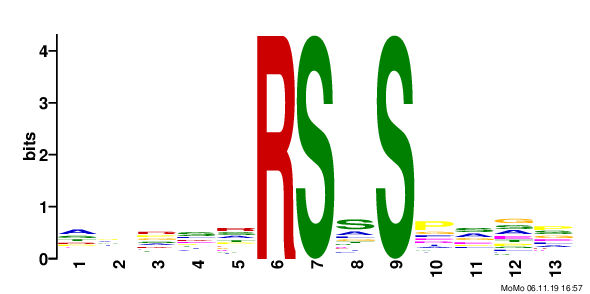 | .....R_S_.S.... | 28.87 | 92 | 2220 | 42 | 3711 | 3.7 |
| 35 | 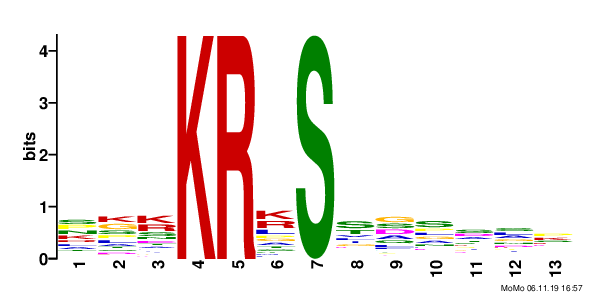 | ...KR._S_...... | 26.32 | 51 | 2128 | 10 | 3669 | 8.8 |
| 36 | 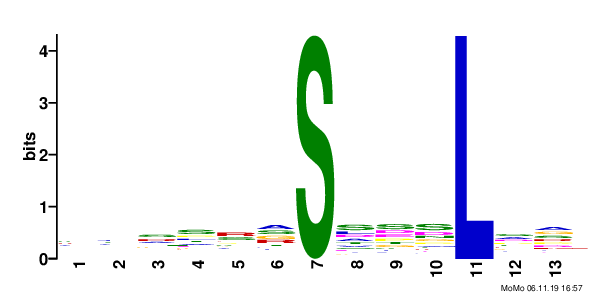 | ......_S_...L.. | 9.42 | 169 | 2077 | 180 | 3659 | 1.7 |
| 37 | 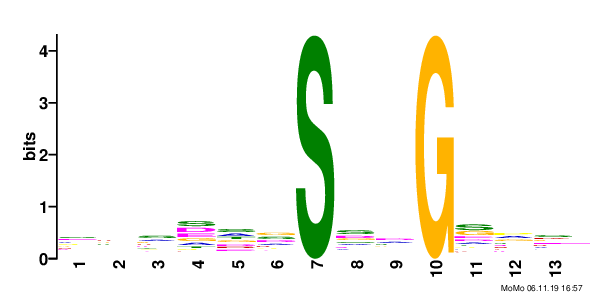 | ......_S_..G... | 9.18 | 235 | 1908 | 285 | 3479 | 1.5 |
| 38 | 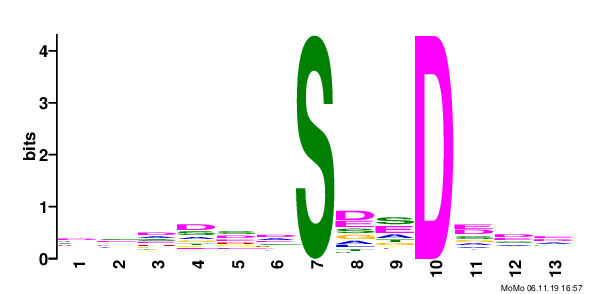 | ......_S_..D... | 8.05 | 214 | 1673 | 275 | 3194 | 1.5 |
| 39 | 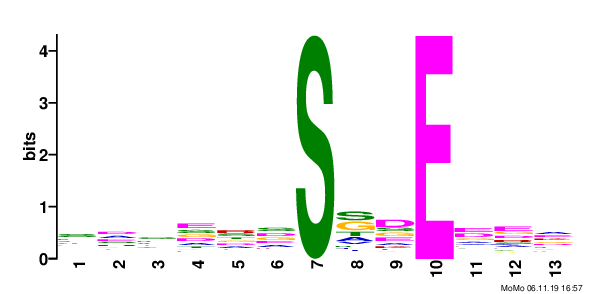 | ......_S_..E... | 7.92 | 149 | 1459 | 184 | 2919 | 1.6 |
| 40 | 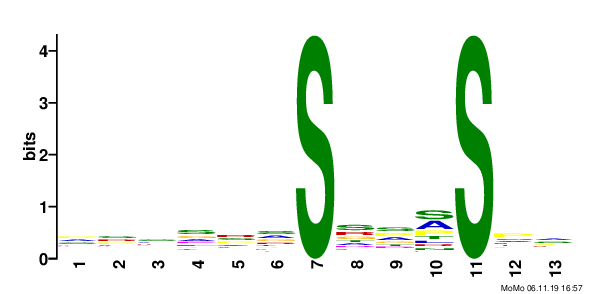 | ......_S_...S.. | 6.16 | 241 | 1310 | 370 | 2735 | 1.4 |
| 41 | 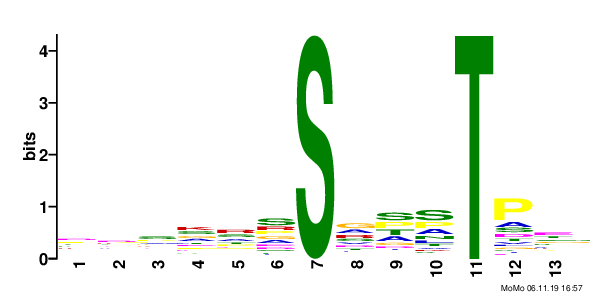 | ......_S_...T.. | 5.33 | 115 | 1069 | 165 | 2365 | 1.5 |
| 42 | 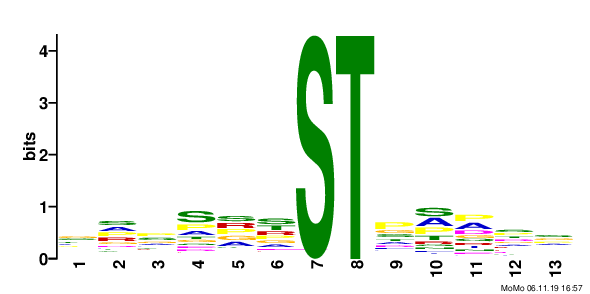 | ......_S_T..... | 6.33 | 108 | 954 | 151 | 2200 | 1.6 |
| 43 | 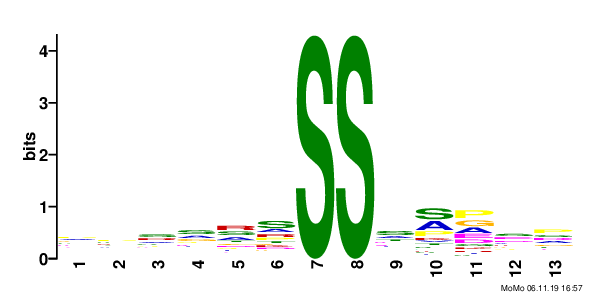 | ......_S_S..... | 4.83 | 182 | 846 | 325 | 2049 | 1.4 |
| 44 | 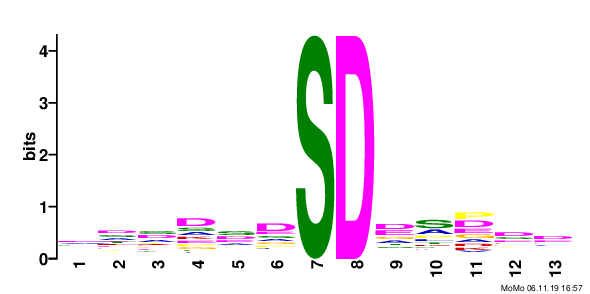 | ......_S_D..... | 5.38 | 116 | 664 | 197 | 1724 | 1.5 |
| 45 | 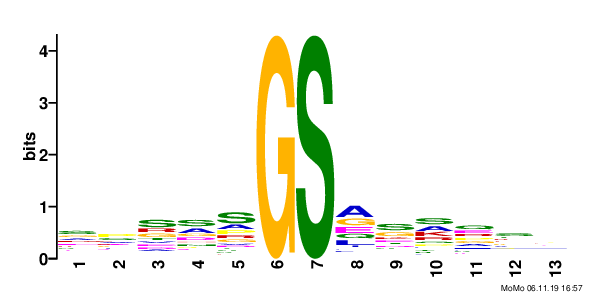 | .....G_S_...... | 3.83 | 73 | 548 | 131 | 1527 | 1.6 |

Note: In the parameter settings, the significance value was not adjusted by Bonferroni correction. A motif-x significance of 0.00018 roughly corresponds to a Bonferroni-corrected P-value of 0.005.
