# Supplementary material for: Hypovirus‐Induced Phosphorylation of CpIre1 Modulates Unfolded Protein Response and Virulence in Cryphonectria parasitica
Source: Mol Plant Pathol. 2026 Feb 15;27(2):e70227. doi: 10.1111/mpp.70227 (PMC12907514; doi:10.1111/mpp.70227)
Supplement: Supplementary file 18 — Table S6: Primers used in this study. [file MPP-27-e70227-s001.docx]

| Primer Name | Sequence （5′to 3′） |
| --- | --- |
| hph-F | CGGTACCCGGGGATCCTCTAG |
| hph-R | GCCTGCAGGTCGACAGAAGATG |
| *CpIre1*-left-F | CCCAAACCCAGAGAGAGACG |
| *CpIre1*-left-R | atatcatcttctgtcgacctgcaggTGGATTGACCGTGGGACTGC |
| *CpIre1-*right-F | tctttctagaggatccccgggtaccgATAGCGGTTCAGGTGGTCAT |
| *CpIre1-*right-R | TCTCGCAGCTCATACAATGG |
| *CpIre1*-all-F | CCCCCCAAAGAAACCCCACA |
| *CpIre1*-all-R | GGCTTCCTCTCGCAGCTCAT |
| *CpIre1*-probe-F | CATCCTTACACAAATAGACCTGGCC |
| *CpIre1*-probe-R | GGTTTTATGAGATGGATGTTGTCGA |
| hyg-proe-F | aaagaaggattacctctaaa |
| hyg-proe-R | ctggaccgatggctgtgtagaa |
| *CpIre1*-com-F | gttaacaagcttgcggccgcGAGGATATGGGATACTTGATGGGC |
| *CpIre1*-com-R | catcttctgtcgacgaattcCGTCGTGTCGAAACCTTGTGAA |
| *CpBip1*-F | ATGAGGCTGTCGCTTTTGGC |
| *CpBip1*-R | TAGGGGTGTTACGGGGAATC |
| *CpHac1^u^* -F | GACAACTCTCAATCTTACTG |
| *CpHac1^u^* -R | TCTACTCGGCGTTGTTCCT |
| *CpHac1*^i^ -F | GAGACATCCGACAAGAAGCCC |
| *CpHac1*^i^ -R | TTGGCCCGTTTTCTGGGAGG |
| RT-18S-F | TCTCGAATCGCATGGCCT |
| RT-18S-R | TTACCCGTTGTAACCACGGC |
| RTQ1 | GTCGACGTAGGATCGTCTAC |
| RTQ2 | TTGCGCAGGACGGTAACACT |
| S896A-F | AGGGCGGCCAGgCGTCATTTGGTGCTACCACCGCC |
| S896A-R | ACCAAATGACGcCTGGCCGCCCTCGAGCTTCTTGCACAG |
| S897A-F | CCAGTCGgCATTTGGTGCTACCACCGCCCACGCGGC |
| S897A-R | ACCAAATGcCGACTGGCCGCCCTCGAGCTTCTTGCACAGG |
| S896A&S897A-F | CGGCCAGgCGgCATTTGGTGCTACCACCGCCCACGCGGC |
| S896A&S897A-F | ACCAAATGcCGcCTGGCCGCCCTCGAGCTTCTTGCACAGG |

**Table S6 Primers used in this study.**
